# Dynamics of Viral Infection and Evolution of SARS-CoV-2 Variants in the Calabria Area of Southern Italy

**DOI:** 10.3389/fmicb.2022.934993

**Published:** 2022-07-28

**Authors:** Carmela De Marco, Claudia Veneziano, Alice Massacci, Matteo Pallocca, Nadia Marascio, Angela Quirino, Giorgio Settimo Barreca, Aida Giancotti, Luigia Gallo, Angelo Giuseppe Lamberti, Barbara Quaresima, Gianluca Santamaria, Flavia Biamonte, Stefania Scicchitano, Enrico Maria Trecarichi, Alessandro Russo, Daniele Torella, Aldo Quattrone, Carlo Torti, Giovanni Matera, Caterina De Filippo, Francesco Saverio Costanzo, Giuseppe Viglietto

**Affiliations:** ^1^Department of Experimental and Clinical Medicine, “Magna Graecia” University, Catanzaro, Italy; ^2^Interdepartmental Center of Services, Molecular Genomics and Pathology, “Magna Graecia” University, Catanzaro, Italy; ^3^UOSD Biostatistics, Bioinformatics, and Clinical Trial Center, IRCCS Regina Elena National Cancer Institute, Rome, Italy; ^4^Department of Health Sciences, “Magna Graecia” University, Catanzaro, Italy; ^5^“Mater Domini” University Hospital, Catanzaro, Italy; ^6^Department of Medical and Surgical Sciences, “Magna Graecia” University, Catanzaro, Italy; ^7^Neuroscience Research Center, “Magna Graecia” University, Catanzaro, Italy

**Keywords:** SARS-CoV-2, variants, surveillance, NGS, immunogenomics

## Abstract

In this study, we report on the results of SARS-CoV-2 surveillance performed in an area of Southern Italy for 12 months (from March 2021 to February 2022). To this study, we have sequenced RNA from 609 isolates. We have identified circulating VOCs by Sanger sequencing of the S gene and defined their genotypes by whole-genome NGS sequencing of 157 representative isolates. Our results indicated that B.1 and Alpha were the only circulating lineages in Calabria in March 2021; while Alpha remained the most common variant between April 2021 and May 2021 (90 and 73%, respectively), we observed a concomitant decrease in B.1 cases and appearance of Gamma cases (6 and 21%, respectively); C.36.3 and Delta appeared in June 2021 (6 and 3%, respectively); Delta became dominant in July 2021 while Alpha continued to reduce (46 and 48%, respectively). In August 2021, Delta became the only circulating variant until the end of December 2021. As of January 2022, Omicron emerged and took over Delta (72 and 28%, respectively). No patient carrying Beta, Iota, Mu, or Eta variants was identified in this survey. Among the genomes identified in this study, some were distributed all over Europe (B1_S477N, Alpha_L5F, Delta_T95, Delta_G181V, and Delta_A222V), some were distributed in the majority of Italian regions (B1_S477N, B1_Q675H, Delta_T95I and Delta_A222V), and some were present mainly in Calabria (B1_S477N_T29I, B1_S477N_T29I_E484Q, Alpha_A67S, Alpha_A701S, and Alpha_T724I). Prediction analysis of the effects of mutations on the immune response (i.e., binding to class I MHC and/or recognition of T cells) indicated that T29I in B.1 variant; A701S in Alpha variant; and T19R in Delta variant were predicted to impair binding to class I MHC whereas the mutations A67S identified in Alpha; E484K identified in Gamma; and E156G and ΔF157/R158 identified in Delta were predicted to impair recognition by T cells. In conclusion, we report on the results of SARS-CoV-2 surveillance in Regione Calabria in the period between March 2021 and February 2022, identified variants that were enriched mainly in Calabria, and predicted the effects of identified mutations on host immune response.

## Introduction

Severe respiratory coronavirus disease 2019 (COVID-19) caused by severe acute respiratory syndrome coronavirus-2 (SARS-CoV-2) started in China in December 2019 (Ren et al., 2020; Zanin et al., 2020; Zhou et al., 2020).

The ancestral strain of SARS-CoV-2 (NC_045512.2), emerged in Wuhan at the end of 2019, was rapidly replaced by a variant lineage named B.1. Moreover, B.1, characterized by a mutation in the gene encoding the spike protein (S protein) at residue 614 (D614), was first detected in Bavaria at the end of January 2020 and subsequently spread globally in March 2020 (https://www.ecdc.europa.eu/en/covid-19/variants-concern) (Volz et al., 2021).

Since then, SARS-CoV-2 have accumulated several mutations leading to the sequential emergence of novel variants. The majority of mutations has occurred in the gene encoding the S protein that mediates viral entry *via* interaction with the receptor for angiotensin-converting enzyme-2 (ACE2) (Letko et al., 2020), particularly in its receptor-binding domain (RBD) (Basu et al., 2021; Kistler and Bedford, 2021).

Mutations in the S protein may facilitate the rapid spreading of virus through mechanisms such as enhanced binding to ACE2 receptor (Lan et al., 2020), counteracting the neutralizing effects of natural antibodies (Piccoli et al., 2020; Hirabara et al., 2021; Fantini et al., 2022) and/or enhancing virus transmissibility to other species (Elaswad et al., 2020; Rodrigues et al., 2020; Luan and Huynh, 2022). For this reason, World Health Organization (WHO), European Centre for Disease Prevention and Control (ECDC), and Istituto Superiore di Sanità in Italy (ISS) have started to monitor emergence and circulation of novel viral variants since January 2020. The current strategies used for tracking SARS-CoV-2 variants include Sanger sequencing of the S gene and next generation sequencing (NGS) of the whole genome (WHO, 2021).

The established nomenclature system for tracking SARS-CoV-2 lineages is based on specific platforms (i.e., GISAID, Nextstrain, Pango). However, letters of the Greek alphabet (i.e., Alpha, Beta, Gamma etc.) have been recommended by the WHO COVID-19 Reference Laboratory Network. Epidemiologically, SARS-CoV-2 variants have been classified as variants under monitoring (VUMs), variants of interest (VOIs), and variants of concern (VOCs) (WHO, 2021). The variants of concern have been associated with increased transmissibility and virulence, altered clinical disease presentation, and reduced vaccine and/or therapeutic efficacy. See definitions at https://www.who.int/en/activities/tracking-SARS-CoV-2-variants.

In the second half of 2020, three different variants classified as VOCs have emerged almost simultaneously. The Alpha variant (B.1.1.7 lineage, also known as 20I/501Y.V1 or VOC 202012/01) was first documented in United Kingdom in September 2020.^1^ It spread quickly first to Europe and then worldwide by mid-December 2020—when it was classified as VOC (Claro et al., 2021; Galloway et al., 2021). Alpha was defined by 19 non-synonymous mutations across its viral genome that included ORF1ab, S, ORF8, and N proteins (Galloway et al., 2021). Notable mutations that characterizes Alpha were Δ69H/70V, N501Y, and P681H (CDC, 2021). The Alpha variant was estimated to be 70–80% more transmissible than the ancestral Wuhan lineage (Stefanelli et al., 2022) although its key mutations appeared to be effectively neutralized by vaccine-induced antibodies (Chen et al., 2021; Tao et al., 2021) and presented also an increase in hospitalization and mortality.

A second variant named Beta (B.1.351 lineage, also known as 20H/501Y.V2 or VOC 202012/02), first identified in South Africa in May 2020, was classified as VOC in December 2020. The Beta VOC presented 21 mutations (CoVariants, 2021), including K417T, E484K, and N501Y (Greaney et al., 2021; Xie et al., 2021).

At the same time, a third variant called Gamma (lineage P.1, also known as 20J/501Y.V3 or B.1.1.28) was documented in travelers from Brazil to Japan in November 2020. It was classified as VOC in January 2021. The Gamma VOC presented 17 amino acid changes, which includes N501Y, E484K, and K417N on the S protein and deletion in ORF1b (Faria et al., 2021; Resende et al., 2021). A related variant (P.2 lineage, also known as B.1.617.2), identified in Brazil and designated as VOC in May 2021, presented the E484K mutation but not the N501Y and K417N changes (Lamarca et al., 2021).

The Delta variant was identified in October 2020 during the third wave of the pandemic in India and was classified as VOC in May 2021. Delta includes three sub-lineages (also known as lineages B.1.617.1, B.1.617.2, and B.1.617.3). In June 2021, Delta lineage B.1.617.2 has become the dominant strain globally. The Delta genome presents more than 20 mutations, including L452R, T478K, and P681R. Compared to the previous variant, and in particular Alpha, Delta presents a marked increase in transmissibility and in hospitalization (Campbell et al., 2021) and in mortality (Fisman and Tuite, 2021).

Finally, a novel variant named Omicron (also known as B.1.1.529) appeared in multiple countries in November 2021, spreading rapidly around the world. The Omicron variant presented more than 60 mutations compared to the ancestral variant (He et al., 2021; Chen et al., 2022; Lupala et al., 2022), many of which had not been observed in other strains. The variant is characterized by 30 amino acid changes, three small deletions, and one small insertion in the S protein, half of which located in the receptor-binding domain of the S protein. Notable mutations that characterize Omicron are N440K, S477N, N501Y, and P681H. Compared to Delta, Omicron presents a marked increase in transmissibility.

In addition to the above described VOCs, many SARS-CoV-2 variants, designated VOIs, including Eta (B.1.525), Iota (B.1.526), Kappa (B.1.617.1), Lambda, (C.37), Epsilon (B.1.429/B.1.427)„ Theta (P.3), and Mu (B.1.621) had also been identified since the beginning, of 2020.^1^

In March 2021, we started the SARS-CoV-2 epidemiological survey in the Calabria Region, Southern Italy. Herein, we report on the prevalence of viral isolates and their dynamic distribution from the beginning of March 2021 to February 2022. Moreover, we employed a bioinformatic tool to investigate the impact of mutations on the immunogenicity of the virus and its potential class I MHC presentation.

## Methods

### Patients

All the data used in this study were previously anonymized as required by the Italian Data Protection Code (Legislative Decree 196/2003) and the general authorizations issued by the Data Protection Authority. The project was approved by the Ethical Committee of Regione Calabria in the Meeting No. 434 held on 16 December 2021. We collected nasopharyngeal swab specimens from 609 patients in the Calabria region. The demographic information is summarized in the Supplementary Table 1.

### Sanger Sequencing

The SARS-CoV-2 oropharyngeal/nasal positive swabs were collected in the hospitals of the Calabria Region. The percentage of sequenced samples was in the range 0.5–5% of registered positive swabs per month. The diagnosis was carried out by the following assays: Allplex SARS-CoV-2 Variants (Arrow Diagnostics Srl, Genoa, Italy), TaqPath COVID-19 high-throughput combo (Thermo Fisher Scientific, USA), Xpert^®^ Xpress SARS-CoV-2 (Cepheid, USA), and cobas^®^ SARS-CoV-2 Test (Roche Diagnostics, USA). Viral RNA was extracted using the NUCLISENS^®^ easyMAG^®^ (bioMérieux, Florence, Italy). The RNA samples with Ct values less than 28 were subjected to retro-transcription through the SuperScript IV VILO master Mix (Invitrogen, Thermofisher Scientific, MA 02451, USA) and then S gene regions were amplified with the primer list reported in the Supplementary Table 2 and Supplementary Figure 1, and using PCR products obtained through the BigDye Direct Cycle Sequencing Kit (Applied Biosystems, Thermofisher Scientific, MA, USA) were then purified according to the instructions of the BigDye Terminator Purification Kit (Applied Biosystems) and sequenced through the Genetic Analyzer 3500 Dx (Applied Biosystems). Electropherograms were analyzed by SeqScape Software, v.4 (Applied Biosystems) and compared to the SARS-CoV-2 reference genome (NC_045512.2).

### Whole-Genome Sequencing

By using the Ion S5™ System (Thermofisher Scientific), WGS was performed. Also, 7 μl of viral RNA were retrotranscribed by using Invitrogen™ SuperScript™ VILO cDNA Synthesis Kit (Thermofisher Scientific). Libraries were prepared using the Ion AmpliSeq SARS-CoV-2 Research Panel (ThermoFisher Scientific), which consists of two primer pools targeting 237 amplicons ranging from 125 to 275 bp in length and tiled across the SARS-CoV-2 genome. Additional five primer pairs targeting human expression controls. Libraries preparation was performed both manually according to the Ion AmpliSeq Library Kit Plus and automatically on the Ion Chef™ Instrument by using the Ion AmpliSeq Kit for Chef DL8 (Thermofisher Scientific) The final concentration of each cDNA library, which was manually prepared, was determined on the Agilent 2100 System by the Agilent High Sensitivity DNA Assay (Agilent Technologies, Santa Clara, CA), following the manufacturer's recommendations. The concentration of cDNA library pool automatically prepared was determined by Ion Library TaqMan Quantitation Kit (Thermofisher Scientific). Barcoded libraries were diluted to 30 pM and then loaded onto the Ion Chef™ Instrument (Thermofisher Scientific) for emulsion PCR, enrichment, and loading onto the Ion S5 520 chip. Post-sequencing run analysis was performed in Ion Torrent Suite Software with the following plugins: The SARS-CoV-2_coverageAnalysis to visualize coverage over targeted regions of SARS-CoV-2 reference genome, COVID19AnnotateSnpEff for variant annotation, and IRMAreport to obtain consensus sequence for each barcode. These 157 SARS-CoV-2 sequence data are available in the GenBank (www.ncbi.nlm.nih.gov) under accession Nos. SUB11466277 and SUB11548142.

### Bioinformatics Analysis

The distribution of mutational cluster in the GISAID database was computed by employing custom R scripting on the results of COVID-miner analysis. The workflow was applied on the multi-FASTA GISAID population available on April 2022. Inter-country resolution bias was smoothed out through use of variation per thousand isolates formula (i.e., VpTI = Mutations^*^1e3) available (Massacci et al., 2020). Phylogenetic analysis was performed using full FASTA database *via* the ETE3 web tool (Huerta-Cepas et al., 2016) on PhyML default parameters (bootstrap: 100).

For the immunogenomic workflow, 36-mer nucleotide sequences were generated for every missense mutation in the S gene (Supplementary Table 3) using a sliding window of three bases to keep the 36-mers in frame. Each mutation resulted in 12 unique nucleotide sequences which were then converted into protein by using EMBOSS transeq program. In total, 516 mutant peptides were generated for subsequent analysis. We used NetMHC-4.0 (Andreatta and Nielsen, 2016) to predict nanomolar (nM) binding affinity of wild-type (Wuhan-H-1 strain, NC_045512.2) or mutated peptides to six alleles of human HLA-A and B, selected among the most frequent alleles present in the Italian Bone Marrow Donor Registry database (Pisanti et al., 2020). We classified the strength of the peptide-MHC interaction for each allele by using the percentile ranks method. Default thresholds were applied. An epitope (wild-type or mutant) was considered as strong binder when its percentile rank was less than or 0.5, while it was considered weak binder when its percentile rank was greater than 0.5 but less than or 2. All peptides that were ranked more than 2 were considered non-binder.

Conversely, the predicted capability of peptides to be recognized by T lymphocytes was assessed by Class I Immunogenicity predictor (http://tools.iedb.org/immunogenicity/) provided by the Immune Epitope Database (IEDB) (Vita et al., 2019).

The accessibility of the peptides on the surface of the S protein was investigated using the PDB database (rcsb.org). To define the exposure score we used the model implemented by Bendell et al. (2014).

The changes in the affinity of Spike–ACE2 interaction exerted by mutations in the S protein was investigated by use of the bioinformatic repository described by Starr et al. (2020). The affinity of binding to ACE2 receptor of mutant residues was expressed as difference in log_10_ of the dissociation constant (K_D_) relative to the that of wild-type residues.

## Results

### Genetic Characterization of SARS-CoV-2 Variants in the Calabria Area

The total number of COVID-19 cases in Calabria from March 2021 to February 2022 (https://www.salute.gov.it) was 138,135. Viral RNA was extracted from 609 isolates by the Microbiology Unit of “Mater Domini” University Hospital of Catanzaro and subsequently sequenced by Sanger method in the Laboratory of Molecular Genomics and Pathology of “Magna Græcia” University of Catanzaro, according to the Italian ISS guideline (www.epicentro.iss.it/en/coronavirus/SARS-CoV-2-integrated-surveillance) since 4 March 2021. Specific regions of the S gene were analyzed to allow identification of circulating VOCs.

**Figure 1:**
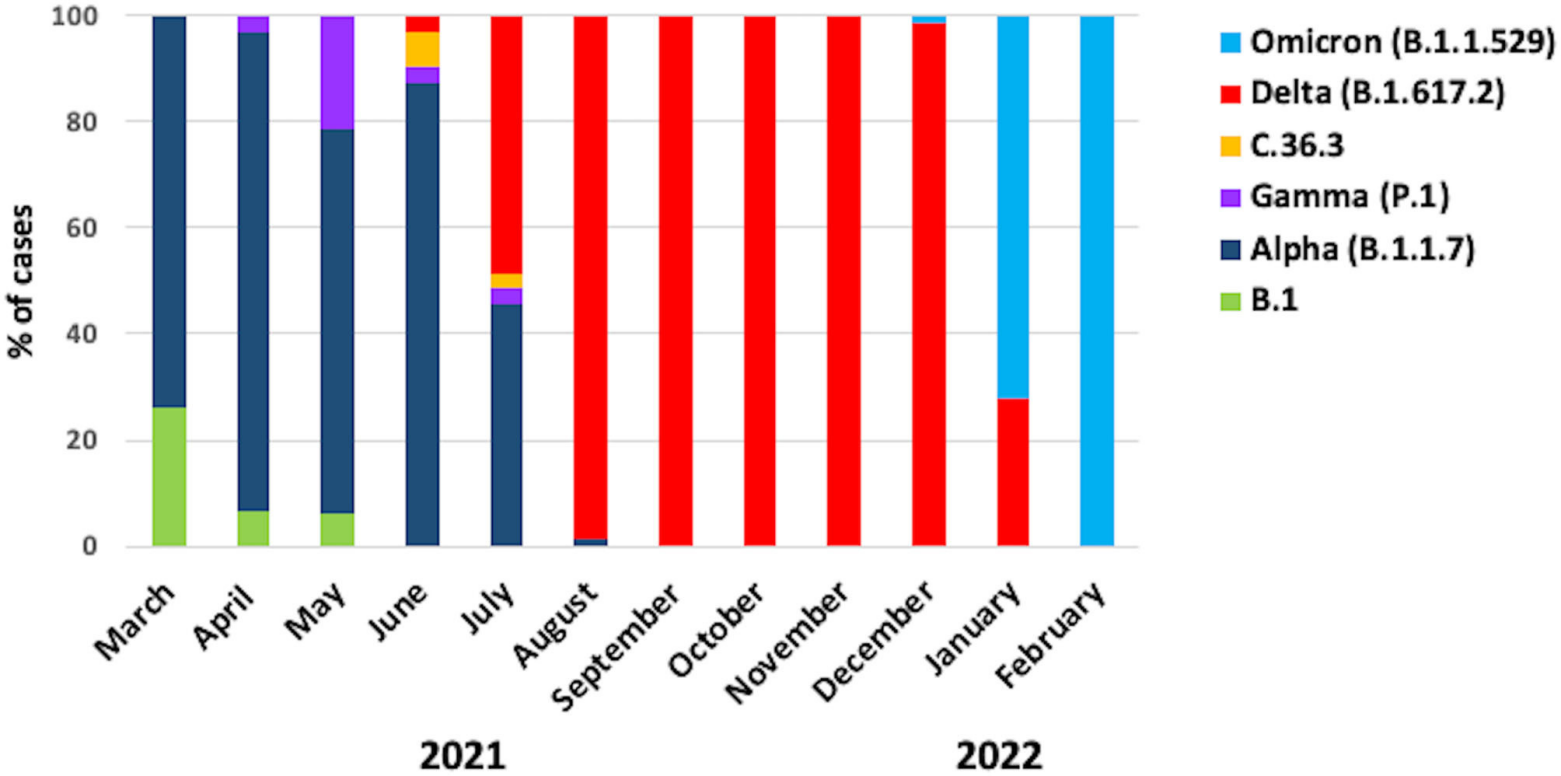
Frequency of the SARS-CoV-2 variants. The graph shows the frequencies of the different variants in the cohort of patients from Regione Calabria in the period between March 2021 and February 2022.

Overall, we found that 4.6% of samples (*n* = 28) were B.1, 33.5% of samples (*n* = 204) were Alpha, 3.1% of samples (*n* = 19) were Gamma, 0.5% of samples (*n* = 3) were C.36.3, 48.3% of samples (*n* = 294) were Delta, and 10% of samples (*n* = 61) were Omicron (Table 1). The graph in Figure 1 shows the distribution of the predominant VOCs in the cohort of Regione Calabria from March 2021 to end of February 2022. In March 2021, B.1 and Alpha were the only circulating lineages, representing 26 and 74% of the sequenced cases, respectively. However, between April 2021 and May 2012, we observed a decrease in the number of B.1 cases and an increase in the number of Gamma cases (6 and 21%, respectively), although Alpha still remained the most common variant (90 and 73%, respectively). In June 2021, we observed a further decrease in the frequency of Gamma (3%) and the disappearance of B.1. At the same time, C.36.3 and Delta variants appeared (6 and 3%, respectively). However, the Alpha variant still remained the most common (87%) one. In July 2021, the dominant VOCs were Alpha and Delta (46 and 48%, respectively), with a smaller number of cases accounted for by C.36.3 and Gamma (3% each). In August 2021, Delta became dominant (99%) resulting the only circulating SARS-CoV-2 variant until the end of December 2021. The first patient infected by the Omicron variant was identified in December 2021, becoming the predominant variant at the end of January 2022 (72%).

**Table 1:** Frequency of VOCs identified in Calabria Region from March 2021 to January 2022.

**Lineage**	**Country first reported**	**Number**	**Percentage (%)**
B.1	NA	28	4.6
Alpha (B.1.1.7)	UK	204	33.5
Delta (B.1.617.2)	India	294	48.3
Gamma (P.1)	Brazil	19	3.1
C.36.3	Egypt	3	0.5
Omicron (B.1.529)	South Africa	61	10

To further characterize the viral isolates, we have analyzed a representative cohort of 157 samples by NGS sequencing of the entire genome of SARS-CoV-2, using the AmpliSeq SARS-CoV-2 Research Panel. The median depth of sequencing was 3,621 (range, 926–24,827), with genome coverage less than 99% in 99% of the viral genome. A median number of 496,246 reads (range 37,344–3,605,323) was generated for each sample (Supplementary Table 4). To analyze NGS results, we generated a consensus sequence for each patient by assembling the viral sequences relative to the reference genome of Wuhan-Hu-1 (NC_045512.2) using the IRMAreport plugin in Ion Torrent Suite. A median value of 46 nucleotide changes/sample was observed (range, 18–58). The NGS analysis identified a total of 256 synonymous and 493 non-synonymous nucleotide changes. The most numerous changes were detected in genes S, ORF1ab, ORF3a, ORF8, and N as shown in Supplementary Figure 2. The complete list of nucleotide changes identified in this study are reported in Supplementary Table 5.

Variant classification based on whole-genome sequences using the Pangolin tool (https://cov-lineages.org/) confirmed the classification based on Sanger sequencing. Among the 157 genomes sequenced by NGS 10 were B.1, 44 were Alpha, 5 were Gamma, 2 were C.36.3, 67 were Delta, and 29 were Omicron, respectively.

**Figure 2:**
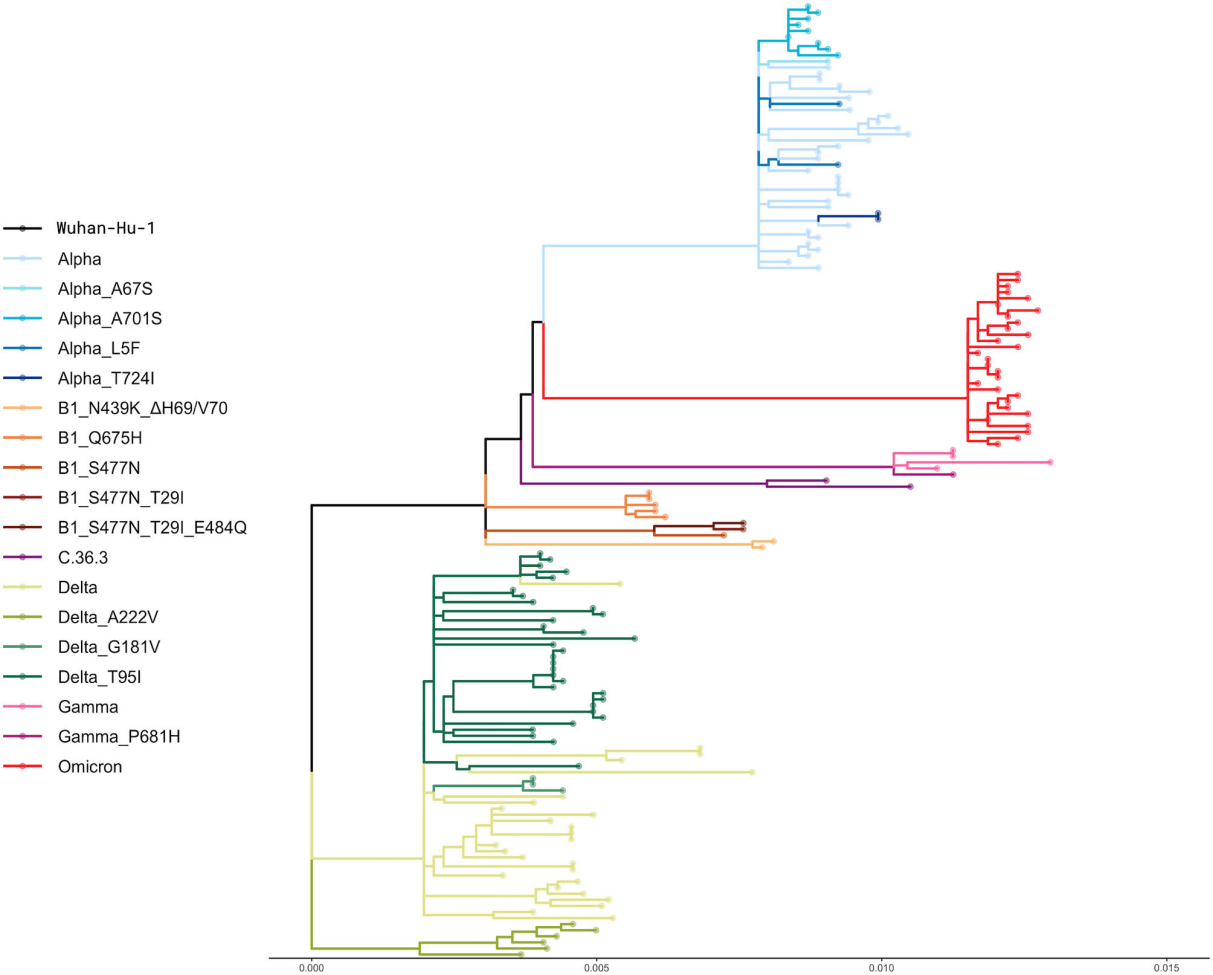
Phylogenetic analysis. The phylogenetic tree shows the genetic relationship between the 157 whole genomes included in this study. A default bootstrap equal to 100 was used. The genetic distance is reported at the bottom.

The phylogeny resulting from the alignment of the 157 complete genomes is shown in Figure 2. However, although all the samples included in this study were collected randomly, some isolates clustered together in small groups of two or three sequences that presented similar genetic distance from the reference sequence (Figure 2).

#### The B.1 Isolates

The B.1 isolates identified by Sanger sequencing were 28. The B.1 isolates, identified from 4 March 2021 until 3 May 2021, were randomly collected from different areas of Calabria according to ISS recommendation.^2^ All B.1 isolates were characterized by D614G in Spike and P4715L in ORF1ab. However, the majority (86%) of B.1 cases identified in Calabria in the Spring 2021 were characterized by the presence of additional mutations, which allowed the definition of the following three sub-genotypes: B1_S477N (*n* =14), B1_Q675H (*n* = 7), and B1_N439K/ΔH69-ΔV70 (*n* = 3) (Table 2). Most of B1_S477N (13/14) samples were characterized also by the presence of T29I; one B1_S477N sample was characterized by the concomitant presence of T29I and E484Q. Most B.1_Q675H isolates (6/7) presented also A222V mutation in the S protein. The last sub-genotype, B1_N439K_ΔH69-ΔV70, presented mutations N439K and ΔH69-ΔV70, a combination that defines lineage B.1.258, a variant emerged in Central Europe since August 2020 (Brejová et al., 2021) and identified in Calabria area in March 2021.

**Table 2:** Mutations identified in VOCs from March 2021 to February 2022.

**Lineage**	**Common spike mutations**	**Sub-genotype specific Spike mutations**	**Additional spike mutations**
B.1	D614G	S477N (14/28)	*T29I (13/14), T29I, E484Q (1/14)*
(*N* = 28)		ΔH69/ΔV70, N439K (3/28)	
		Q675H (7/28)	*A222V (6/7)*
Alpha (B.1.1.7)	ΔH69/V70, ΔY144, N501Y, A570D, D614G, P681H, T716I, S982A,	L5F (3/204)	*S13I (1/3)*
(*N* = 204)	D1118H	A701S (10/204)	*T618I (1/10)*
		T724I (4/204)	
		A67S (1/204)	
Gamma (P.1) (*N* = 19)	L18F, T20N, P26S, D138Y, R190S, K417T, E484K, N501Y, D614G, H655Y, T1027I, V1176F	P681H (1/19)	
C.36.3 (*N* = 3)	D614G, Q677H		
Delta (B.1.617.2)	T19R, E156G, ΔF156/R157, R158G, L452R, T478K, D614G, P681R,	T95I (122/294)	*Q677H/P (6/122)*
(*N* = 294)	D950N	A222V (32/294)	*Y145H (1/32)*
		G181V (7/294)	
Omicron (B.1.1.529)	A67V, ΔH69/V70, ΔG142/Y145insI, ΔN211/L212insI,	R346K (46/61)	
(*N* = 61)	R214/215insEPE, G339D, S371L, S373P, S375F, K417N, N440K, G446S, S477N, T478K, E484A, Q493R, T547K, D614G, H655Y, N679K, P681H, N764K, D796Y, N856K, Q954H, N969K, L981F	A701V (9/61)	

**Figure 3:**
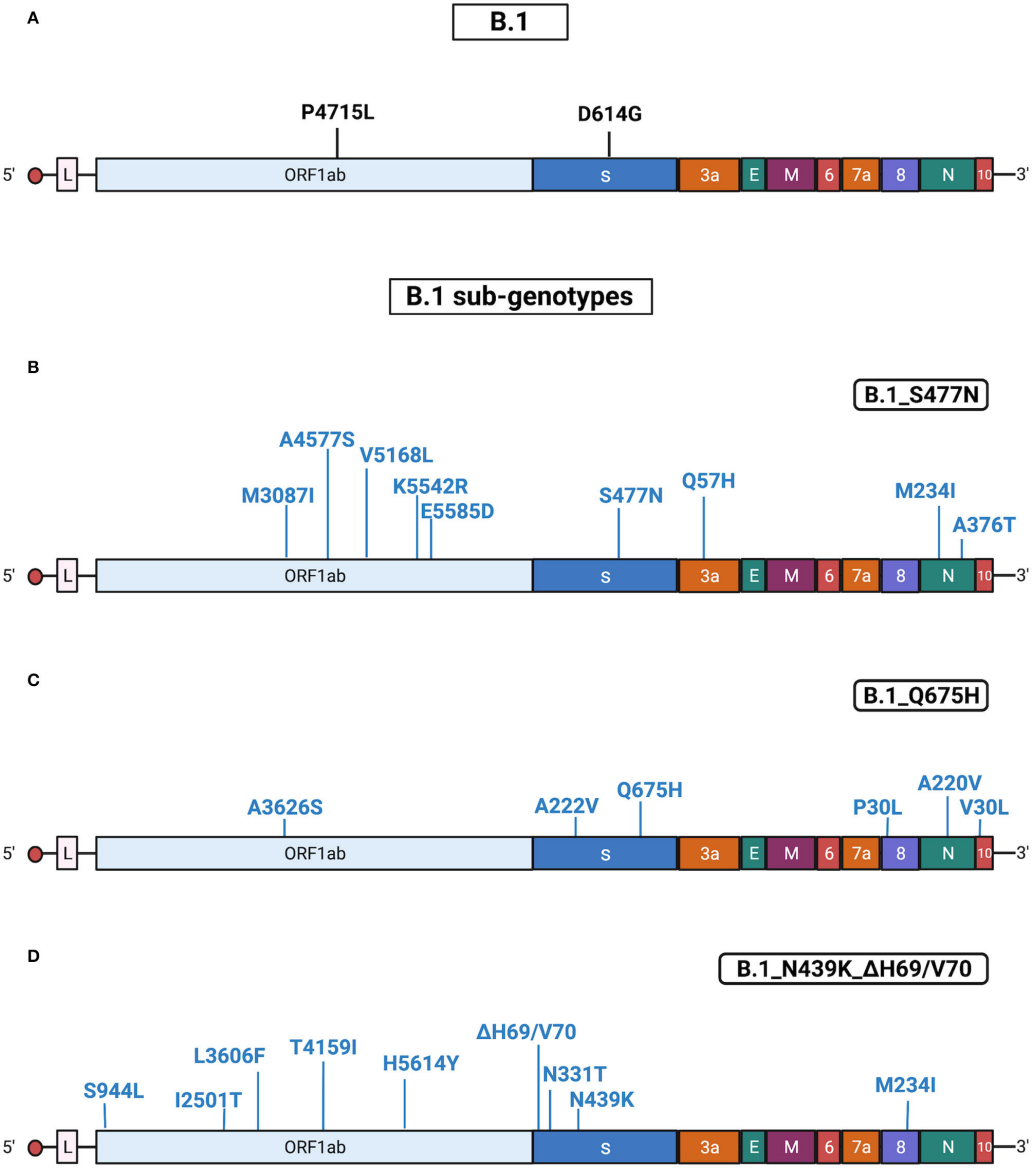
Substitutions in B.1 isolates. The figure shows the substitutions identified in the B.1 isolates by NGS. Color code: black, substitutions common to all B.1 isolates; blue, substitutions specific for the different B.1 sub-genotypes. **(A)** Substitutions common to all B.1 isolates. **(B)** Substitutions identified in all B.1 isolates characterized by the presence of S477N (sub-genotype B.1_S477N). **(C)** Substitutions identified in all B.1 isolates characterized by the presence of Q675H (sub-genotype B.1_Q675H). **(D)** Substitutions identified in all the B.1 isolates characterized by the presence of N439K/ΔH69-ΔV70 (sub-genotype B.1_N439K/ΔH69-ΔV70). Gene abbreviations: ORF, open reading frame; S, Spike; E, envelope; M, membrane; N, nucleocapsid. Adapted from “Genome Organization of SARS-CoV” by BioRender.com (2022). Retrieved from https://app.biorender.com/biorender-templates.

Results obtained with Sanger sequencing were confirmed by NGS analysis of 10 representative samples distributed among the different sub-genotypes. A complete list of mutations identified in B.1 isolates is reported in Supplementary Tables 5, 6. See also Figure 3 for a graphical representation of B.1 sub-genotypes.

#### Alpha Isolates

Alpha isolates identified by Sanger sequencing were 204. Alpha variants in Regione Calabria were isolated from 3 March 2021 until 8 August 2021 from different areas of Calabria according to ISS recommendation.^2^ All sequenced Alpha isolates presented a similar pattern of mutations and/or deletions in Spike (ΔH69/V70, ΔY144, N501Y, A570D, D614G, P681H, T716I, S982A, and D1118H), ORF1ab (T1001I, A1708D, I2230T, P4715L, and Δ3676/3678), ORF8 (Q27^*^, R52I, and Y73C), and N (D3L, R203K, G204R, and S235F) proteins.

We found that 8% of Alpha samples presented additional specific mutations in S protein that allowed us to define three different sub-genotypes: Alpha_A701S (*n* = 10), Alpha_T724I (*n* = 4) and Alpha_L5F (*n* = 3) (Table 2). Notably, one patient infected by the Alpha in July 2021 presented the T95I mutation in the S protein, which appeared later during the emergence of Delta.

**Figure 4:**
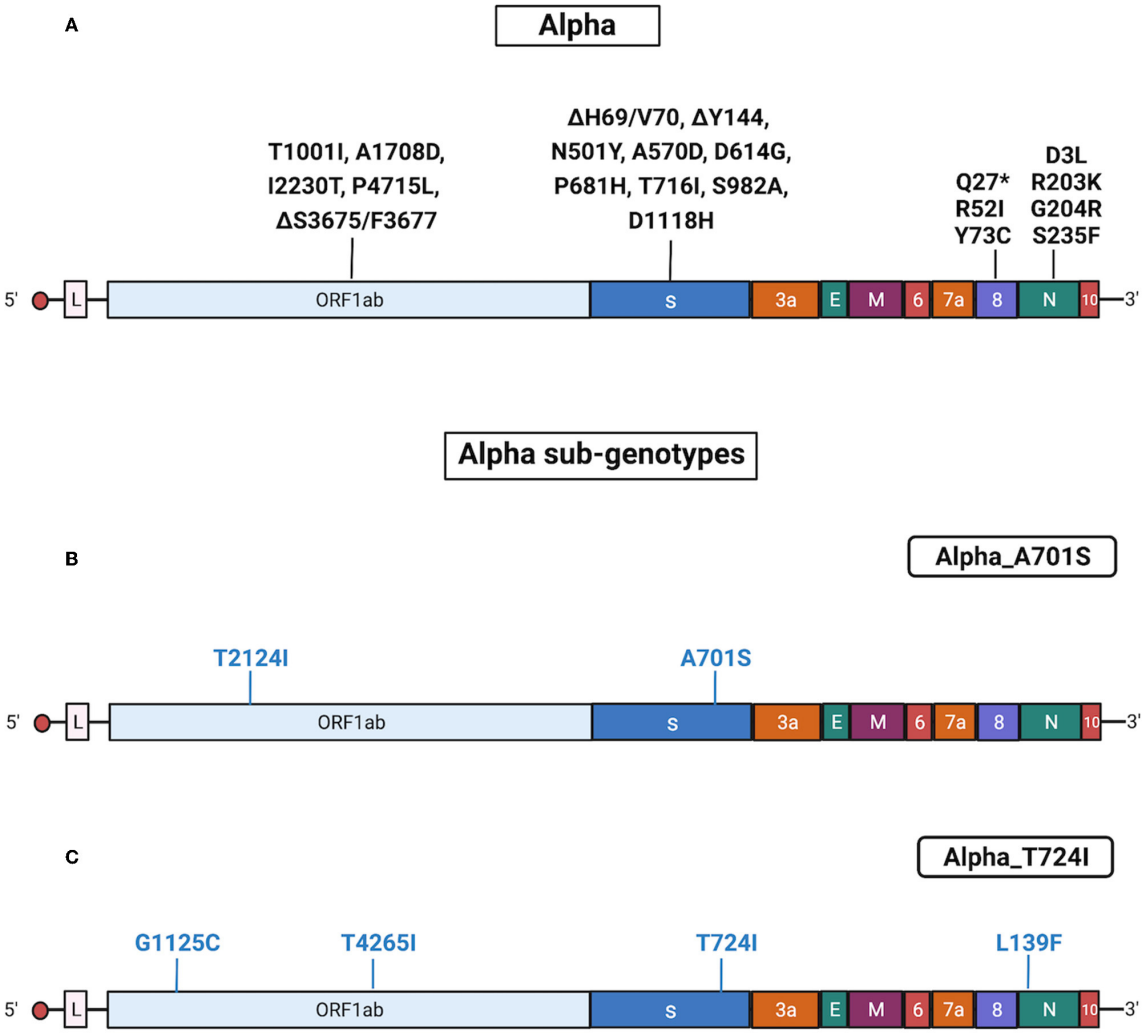
Substitutions in Alpha isolates. The figure shows the substitutions identified in the Alpha isolates by NGS. Color code: black, substitutions common to all Alpha isolates; blue, substitutions specific for the different Alpha sub-genotypes. **(A)** Substitutions common to all Alpha isolates. **(B)** Substitutions identified in all Alpha isolates characterized by the presence of A701S (sub-genotype Alpha_A701S). **(C)** Substitutions identified in all Alpha isolates characterized by the presence of T724I (sub-genotype Alpha_T724I). Gene abbreviations: ORF, open reading frame; S, Spike; E, envelope; M, membrane; N, nucleocapsid. Adapted from “Genome Organization of SARS-CoV” by BioRender.com (2022). Retrieved from https://app.biorender.com/biorender-templates.

Results obtained with Sanger sequencing were confirmed by NGS of 44 representative samples distributed among the different sub-genotypes. A complete list of mutations identified in Alpha isolates is reported in Supplementary Tables 5, 6. Also, see Figure 4 for a graphical representation of Alpha sub-genotypes.

#### Gamma and C.36.3 Isolates

Gamma isolates (*n* = 19) identified by Sanger sequencing were collected from 27 April 2021 until 7 July 2012. Gamma samples were collected randomly from different areas of the Regione Calabria according to ISS recommendation.^2^ All Gamma isolates had a common pattern of mutations in Spike (L18F, T20N, P26S, D138Y, R190S, K417T, E484K, N501Y, D614G, H655Y, T1027I, and V1176F), ORF1ab (S1188L, K1795Q, P4715L, E5665D, and ΔS3675/F3677), ORF3a (S253P), ORF8 (E92K), and N (R203K, G204R, and P80R) proteins.

One Gamma isolate presented an additional mutation in S protein, P681H, located near the critical furin cleavage site, which will appear later during the emergence of Delta as P681R and of Omicron as P681H.

In June 2021, three patients were infected with a rare variant, named C.36.3, that had appeared in North Italy in May 2021 and was characterized by lower sensitivity to antibodies (Castelli et al., 2021). The C.36.3 samples subjected to the NGS showed common mutations in the genes encoding Spike (S12F, Δ69/70, W152R, R346S, L452R, D614G, Q677H, and A899S), ORF1ab (E102K, A859V, T1246I, D1639N, P2287S, D2980N, D3222N, G3278S, S3687L, L3691S, T4090I, P4715L, A4921V, and D5429Y), and N (c.-3delA, R203K, G204R, and G212V) proteins. This lineage was characterized by the L452R mutation that, later, will become typical of Delta. The results obtained with Sanger sequencing were confirmed by NGS sequencing of five representative samples. A complete list of mutations identified in Gamma and in C.36.3 isolates is reported in Supplementary Tables 5, 6. Also, see Supplementary Figure 3 for a graphical representation of C.36.3 mutations.

#### Delta Isolates

Delta isolates (*n* = 294) were identified by Sanger sequencing from 30 June 2021 until 13 January 2022. Delta samples were randomly collected from different areas of Regione Calabria according to ISS recommendation.^2^ This VOC became the predominant variant in Calabria in September 2021, displacing all other VOCs, probably because of its increased transmissibility (Moghaddar et al., 2021) and lower sensitivity to neutralization by antibodies (Planas et al., 2021). The sequenced Delta samples had common mutations in the genes encoding Spike (T19R, G142D, 156del, 157del, R158G, L452R, T478K, D614G, P681R, and D950N), ORF1ab (P4715L, G5063S, and P5401L), ORF3a (S26L), ORF7a (T120I and V82A), M (I82T), and N (R203M, D377Y, and D63G) proteins.

As indicated above, originally, Delta included three different sub-lineages (B.1.617.1, B.1.617.2, and B.1.617.3), though B.1.617.2 became dominant worldwide in June 2021. Many B.1.617.2 genomes were classified as AY by Pangolin analysis. In Italy, all AY subtype of Delta variants emerged at the beginning of Summer 2021, peaked in August and disappeared in the Autumn. The AY isolates identified in the Calabria area were distributed among several sub-lineages: AY.4 (30.7%); AY.7.2 (13.4%); AY.43 (13.4%); AY.42 (*n* = 5); AY.61 and AY.122 (9.6%); AY.46 (5.7%); AY.122.1 and AY.102 (3.8%); AY.58, AY.20, AY.125, AY.39.2, AY.34, AY.98.1, and AY.23 (1.9%). In addition, we found that 55% (161/294) of Delta samples presented additional specific mutations in S protein, allowing us to define three different sub-genotypes: Delta_T95I (*n* = 122), Delta_A222V (*n* = 32) and Delta_G181V (*n* = 7; Table 2).

**Figure 5:**
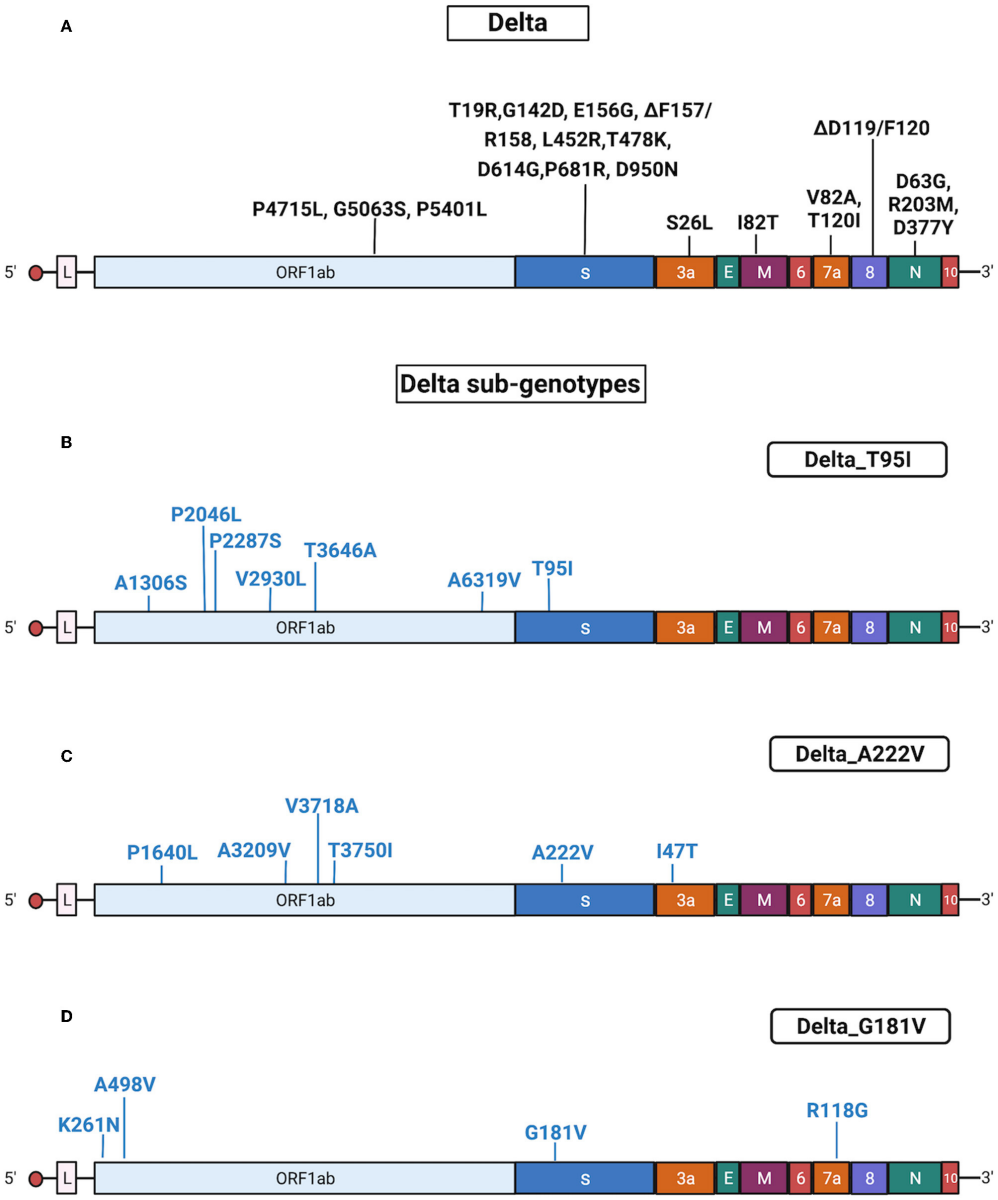
Substitutions in Delta isolates. The figure shows the substitutions identified in the Delta isolates by NGS. Color code: black, substitutions common to all Delta isolates; blue, substitutions specific for the different Delta sub-genotypes. **(A)** Substitutions common to all Delta isolates. **(B)** Substitutions identified in all Delta isolates characterized by the presence of T95I (sub-genotype Delta_T95I). **(C)** Substitutions identified in all Delta isolates characterized by the presence of A222V (sub-genotype Delta_A222V). **(D)** Substitutions identified in all Delta isolates characterized by the presence of G181V (sub-genotype Delta_ G181V). Gene abbreviations: ORF, open reading frame; S, Spike; E, envelope; M, membrane; N, nucleocapsid. Adapted from “Genome Organization of SARS-CoV” by BioRender.com (2022). Retrieved from https://app.biorender.com/biorender-templates.

The results obtained by Sanger sequencing were confirmed by NGS sequencing of 67 representative samples. A complete list of mutations identified in Delta isolates is reported in Supplementary Tables 5, 6. See also Figure 5 for a graphical representation of Delta sub-genotypes. Interestingly, AY.42 was similar to the Delta_T95I sub-genotype, differing from classic Delta only for the presence of T95I. Conversely, the AY.61 lineage presented a set of mutations similar to the Delta_A122V sub-genotype (Supplementary Table 5).

#### Omicron Isolates

Omicron isolates (*n* = 61) were identified by Sanger sequencing from 20 December 2021 in the samples collected from different areas of Calabria. To our knowledge, Omicron samples were collected randomly according to ISS recommendation.^2^

**Figure 6:**
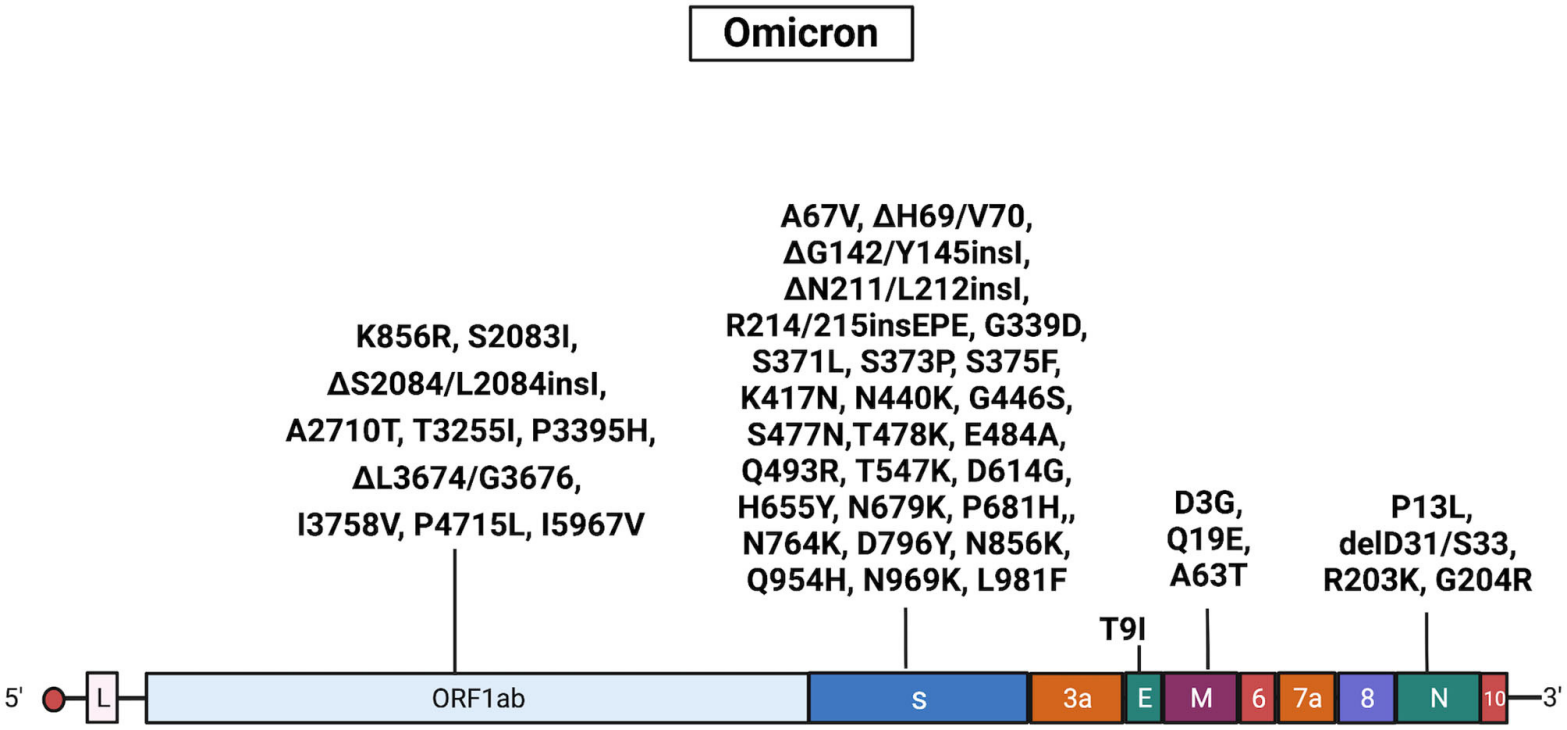
Mutations in Omicron isolates. The figure shows the substitutions identified in the Omicron isolates by NGS. Black, substitutions common to all Omicron isolates. Gene abbreviations: ORF, open reading frame; S, Spike; E, envelope; M, membrane; N, nucleocapsid. Adapted from “Genome Organization of SARS-CoV” by BioRender.com (2022). Retrieved from https://app.biorender.com/biorender-templates.

The Omicron samples had common mutations in the genes encoding the Spike (A67V, ΔH69/V70, ΔG142/Y145insI, ΔN211/L212insI, R214/215insEPE, G339D, S371L, S373P, S375F, K417N, N440K, G446S, S477N, T478K, E484A, Q493R, T547K, D614G, H655Y, N679K, P681H, N764K, D796Y, N856K, Q954H, N969K, and L981F), ORF1ab (K856R, S2083I, ΔS2084/L2084, A2710T, T3255I, P3395H, ΔL3674/G3676, I3758V, P4715L, and I5967V), E (T9I), M (D3G, Q19E, and A63T), and N (P13L, ΔD31/S33, R203K, and G204R) proteins. Results obtained with Sanger sequencing were confirmed by NGS sequencing of 29 representative samples. A complete list of mutations identified in Omicron isolates is reported Supplementary Tables 5, 6. Also, see Figure 6 for a graphical representation of Omicron mutations.

### Distribution of Sub-genotypes

**Figure 7:**
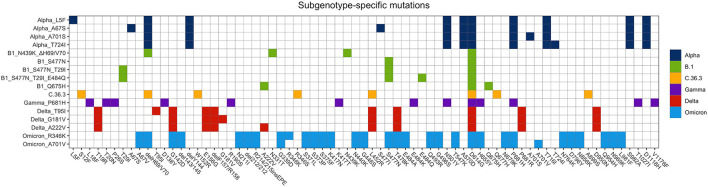
Distribution of mutations identified in the different variants' sub-genotypes. The chart reports the mutations that characterize the different variants' sub-genotypes identified in this study.

**Figure 8:**
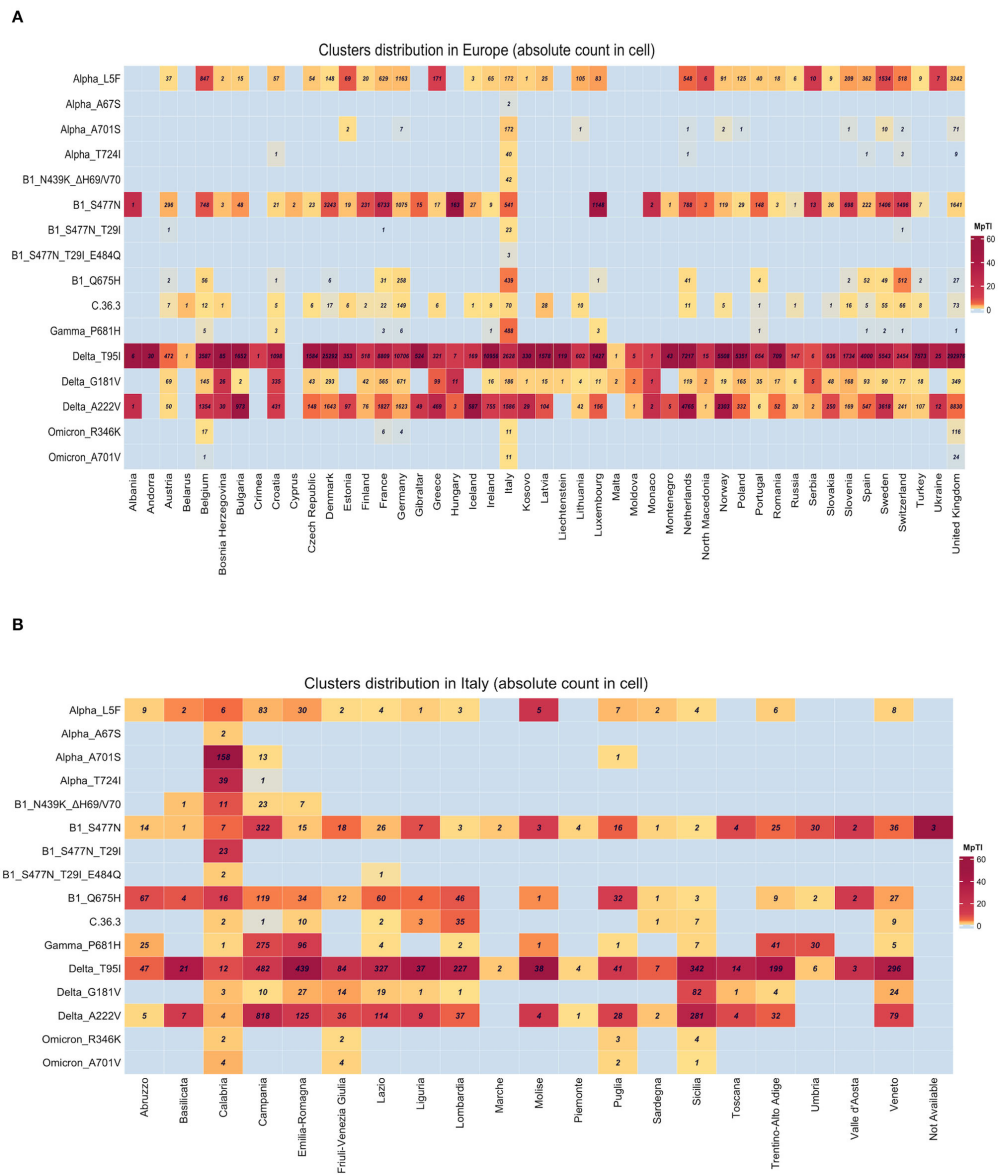
Geographical distribution of the different variants' sub-genotypes. Heatmaps show the distribution of the different sequences available in GISAID database. Numbers within the boxes represent the absolute numbers of sub-genotypes characterized by the presence of the mutations indicated on the left side among European countries **(A)** or Italian regions **(B)**. Variations per Thousand Isolates more than 10 are plotted.

The sequencing analysis of viral isolates included in this study allowed us to identify sub-genotypes of the six main variants sequenced (B.1, Alpha, Gamma, C.36.3, Delta, and Omicron) that were characterized by specific combinations of mutations (Figure 7). The distribution of such sub-genotypes was investigated in Europe, Italy and in Calabria (Figures 8A,B, respectively), through an automated analysis of the GISAID database (https://covid-miner.ifo.it/app/home).

From the analysis of the 10 million sequences present in the database we found that some of the sub-genotypes identified in this study (Alpha_L5F, B1_S477N, Delta_T95, and Delta_G181V, Delta_A222V) were largely distributed among the majority of European countries.

On the other hand, the analysis of the distribution of viral variants showed that a few genotypes were mostly present in Italy, even when normalizing by the inter-country sequencing resolution bias (variation per 1,000 isolates is more than 10). For example, the majority of B1_N439K_DH69/V70, Alpha_T724I, and Alpha_A701S variants' sequences were reported in Italy (Figure 8A).

Finally, a more detailed analysis of viral distribution across Italian regions showed that some variants (i.e., B1_Q675H, B1_S477N, and Delta_T95I) were distributed homogeneously across all regions, with less than 90% of coverage, whereas other variants such as B1_S477N_T29I_E484Q, Alpha_A701S, and Alpha_T724I were enriched predominantly in the Calabria area (frequency of 90–100%). See Figure 8B.

### Immunogenomic Analysis

The emergence of viral variants that were more contagious and/or partially resistant to antibodies has suggested that some widespread mutations may have deleterious effects on virus recognition by host immune system. For this reason, we have investigated the immunological impact of specific combinations of mutations, as identified in this study, performing a multi-layered immunogenomic bioinformatic analysis described in the Section Materials and Methods.

First, we investigated whether specific mutations created neo-epitopes that presented differential capability to bind class I MHC alleles. To this aim, we measured the binding affinity toward different class I MHC alleles of *in silico*-generated peptides carrying wild-type or mutant amino acids identified in the different sub-genotypes in this study (Table 2).

The analysis was performed by interrogating 6,204 unique peptide-class I MHC pairs present in the database. As a result, 28 peptides were classified as strong MHC binders (percentile rank ≤ 0.5) while 106 peptides were classified as weak MHC binders (0.5 < percentile rank ≤ 2). Among the 28 peptides with high predicted binding affinity to MHC, 13 peptides were of wild-type while 15 peptides were of mutant, as indicated in Supplementary Table 3. Notably, the wild-type peptides whose mutant counterparts presented predicted weak or completely non-binding score to MHC were 5 (Table 3). Eight peptides were strong binders both in wild-type and in mutated forms.

**Table 3:** Binding affinity of peptides to class I MHC.

**Mutation**	**WT**	**Mutant**	**Class I MHC**	**Rank WT**	**Rank MUT**	**Binder WT**	**Binder MUT**
A701S	AENSVAYSNNSI	SENSVAYSNNSI	HLA-B4403	0.4	0.6	Strong	Weak
T29I	PAYTNSFTRGVY	PAYINSFTRGVY	HLA-A0101	0.06	0.6	Strong	Weak
T29I	YTNSFTRGVYYP	YINSFTRGVYYP	HLA-A0101	0.08	0.7	Strong	Weak
T19R	NLTTRTQLPPAY	NLRTRTQLPPAY	HLA-A0101	0.15	3	Strong	None
T19R	LTTRTQLPPAYT	LRTRTQLPPAYT	HLA-A0101	0.4	9	Strong	None
G142D	FQFCNDPFLGVY	FQFCNDPFLDVY	HLA-A0101	0.6	0.4	Weak	Strong
D138Y	KVCEFQFCNDPF	KVCEFQFCNYPF	HLA-B1801	1.1	0.2	Weak	Strong
D138Y	KVCEFQFCNDPF	KVCEFQFCNYPF	HLA-B4403	0.9	0.4	Weak	Strong
D138Y	VCEFQFCNDPFL	VCEFQFCNYPFL	HLA-B4403	1	0.5	Weak	Strong
D138Y	CEFQFCNDPFLG	CEFQFCNYPFLG	HLA-B4403	0.9	0.4	Weak	Strong
R190S	LREFVFKNIDGY	LSEFVFKNIDGY	HLA-A0101	4	0.175	None	Strong
K417T	PGQTGKIADYNY	PGQTGTIADYNY	HLA-A0101	1.3	0.5	Weak	Strong

As a consequence of the method used to generate the peptides, a single residue was included into multiple peptides so that the mutations were less numerous than peptides. In particular, the mutations in the S protein that predicted weaker or non-binding to class I MHC were T29I (B.1), A701S (Alpha), and T19R (Delta) (Supplementary Table 3 and Table 3). Conversely, the mutations in the S protein that potentially led to increased MHC binding were G142D (Delta), D138Y (Gamma), R190S (Gamma), and K417T (Gamma) (Supplementary Table 3 and Table 3).

Subsequently, we performed an immunogenomic analysis to predict the capability of peptide recognition by T lymphocytes, to determine the impact of specific mutations on T cell recognition and/or activation. The capability of peptide recognition by T lymphocytes was classified using a delta value calculated according to changes in the predicted binding of mutant peptide-MHC pair to T cells compared to wild-type peptide-HLA pair.

**Figure 9:**
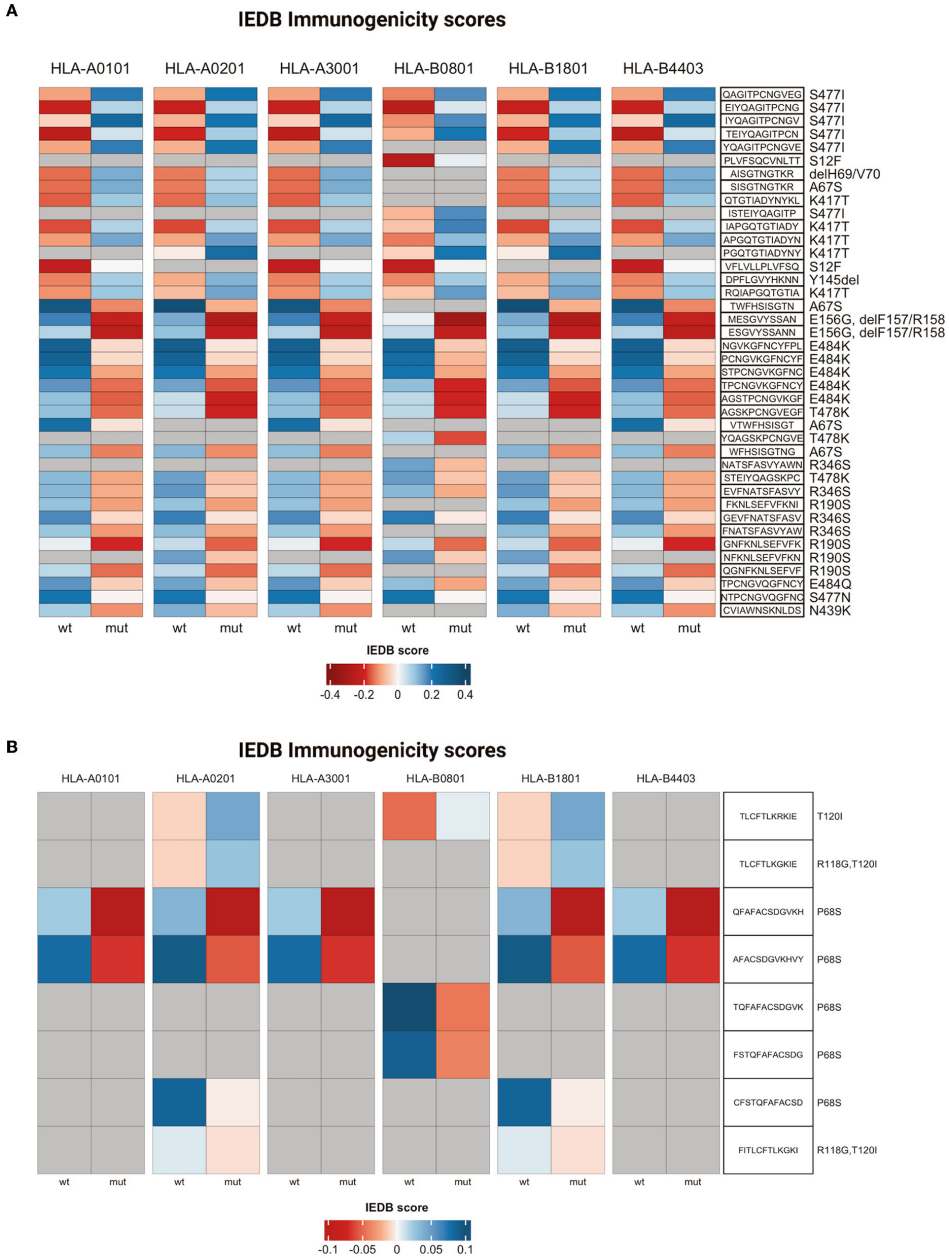
T cell immunogenicity analysis. Heatmaps representing T cell immunogenicity scores for wild-type and mutated peptides from Spike **(A)** or ORF7a **(B)**. IEDB scores range from dark red (low immunogenicity) to blue (high immunogenicity).

The analysis was performed by interrogating 6,204 peptide-class I MHC pairs present in the database. The results of this analysis indicated that 195 (3.1%) mutant peptide-MHC pairs presented a negative immunogenic score compared to the corresponding wild-type pairs. The 195 peptides present in the MHC pairs with a negative immunogenic score derived from 16 different mutations. Notably, the mutations that induced a shift toward a negative immunogenic score with a delta value less than 0.2 included A67S present in Alpha, E156G, ΔF157/R158, and T478K present in Delta, R346S present in C.36.3, N439K, S477N, and E484Q present in B.1, R190S, and E484K present in Gamma (Figure 9A and Supplementary Table 7).

Conversely, 236 (3.8%) mutant peptide-MHC pairs presented a positive immunogenic score compared to the corresponding wild-type pairs and were generated by 24 different mutations (Supplementary Table 7). The mutations that induced a shift toward a positive immunogenic score with a delta value more than or 0.2 included A67S, S477I, ΔH69/V70 (Alpha), K417T (Gamma), and S12F (C.36.3) (Figure 9A and Supplementary Table 7).

Finally, five mutations (A67S, ΔH69/V70, delY145, S477N, and L18F) gave origin to peptides/MHC pairs with both a positive and negative immunogenic score. In the case of A67S peptides with a negative score were 5, while those with a positive score were 21 (Supplementary Table 7).

Also, the mutations in ORF7a protein apparently altered the binding affinity to class I MHC and/or T cell recognition (Supplementary Table 8). In fact, the analysis performed in this study indicated that 16 out of 384 (4.2%) ORF7a peptide-MHC pairs predicted decreased immunogenicity in comparison with their wild-type counterparts included the P68S mutation present in Delta variant (Figure 9B and Supplementary Table 8).

### Accessibility and Receptor-Binding Properties of Identified Mutants

**Figure 10:**
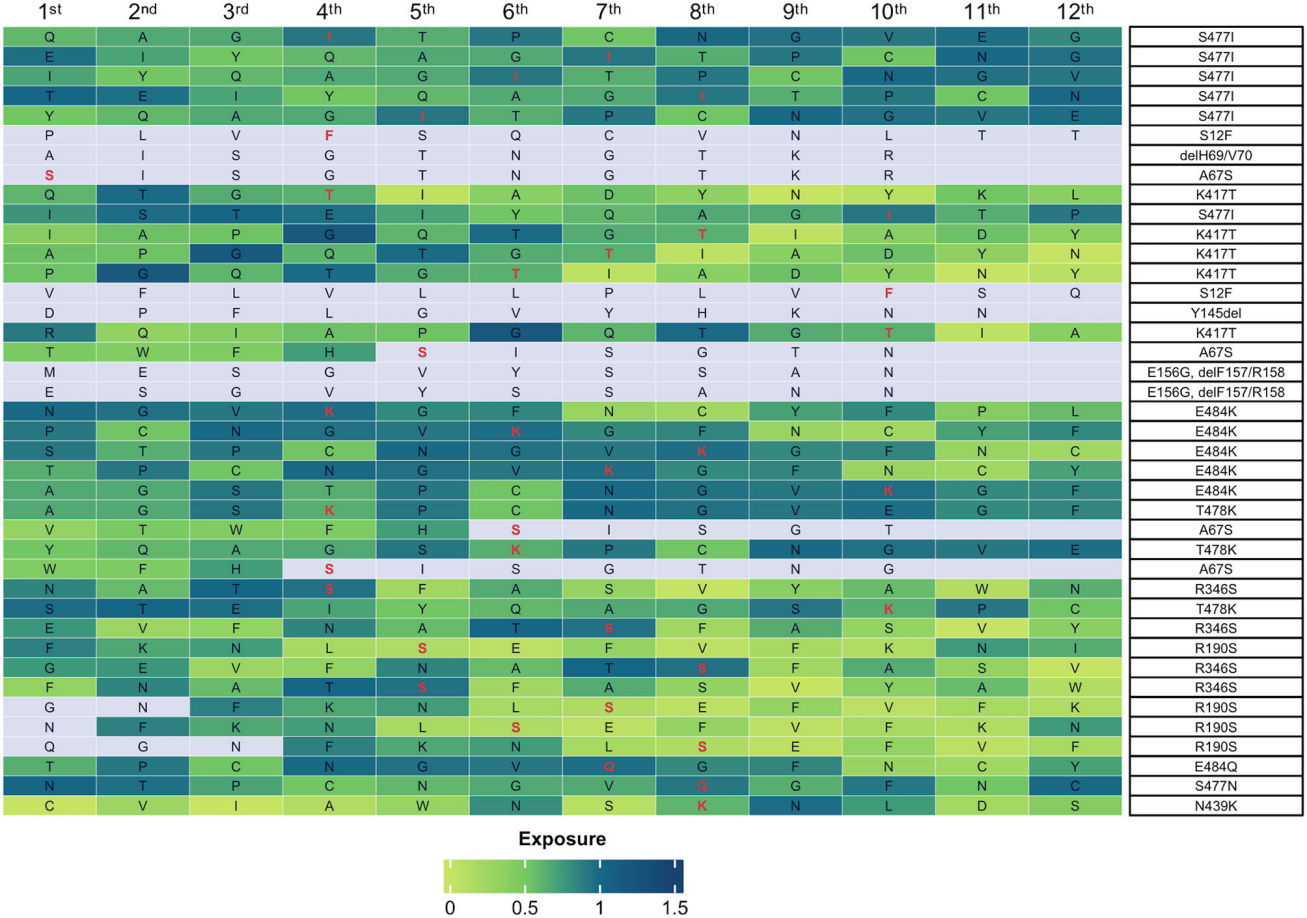
Accessibility of mutant peptides on surface of the S protein. The heatmap shows the accessibility scores of peptides containing the residues indicated on the right. Boxes represent the amino acid residue under analysis. The mutations present in the sub-genotypes identified in this study are highlighted in red. The accessibility score is indicated by the shades of green on the scale from 0 (light green, no exposure) to 1.5 (dark green, high exposure). The color gray indicates that no exposure score is available.

Subsequently, to provide additional information for identified mutations, we investigated the accessibility on the surface of the S protein of the epitopes containing the residues that were subjected to mutations. To this aim, we exploited the data present in the Protein Data Bank (PDB; http://www.rcsb.org/pdb/) (Berman et al., 2000). First, we identified the residues for which accessibility scores were available within the database and determined the accessibility scores. The results showed that 40 peptides corresponding to 12 residues presented a score greater than 0.25 and thus were considered accessible (Bendell et al., 2014; Figure 10).

In most cases, the mutations occurred in residues contained within the receptor-binding domain (RBD) of the S protein (R346S, K417T, N439K, S477I/N, T478K, and E484K/Q). In the remaining cases, the mutations occurred in residues contained within the NTD domain (S12F, A67S, ΔH69/V70, E156GΔF157/R158, and R190S). Notably, the highest accessibility score was generated by mutations E484Q/K (score, 0.968), R346S (score, 0.947), and S477I (score, 0,919). Also, see Supplementary Table 9.

Finally, we investigated the impact of the identified mutations on the interaction between the RBD domain of the S protein and ACE2 receptor. To this aim, we accessed the raw data described in Starr et al. (2020), which were used to map the mutations identified in this study. Eleven mutations identified in this study were present in the database. For each of the 11 mutations present in the database, the shift in free energy caused by the codon change was extracted (Supplementary Table 10). The results of this analysis showed that the mutation that caused the highest increase in binding affinity for ACE2 was N501Y (0.24 Δlog_10_KD). Increased binding affinity was also caused by E484Q (0.03 Δlog_10_KD) and E484K (0.06 Δlog_10_KD). The replacement of the serine at position 477, with an asparagine (S477N), results in an increased binding affinity (0.06 Δlog_10_KD) whereas the replacement of the serine with an isoleucine (S477I) decreases the binding affinity for ACE2 (−0.06 Δlog_10_KD). In contrast, the K417N causes the greatest decrease in binding affinity for ACE2 (−0.26 Δlog_10_KD).

## Discussion

In this study, we report on the results of SARS-CoV-2 surveillance performed in the Calabria area (Southern Italy) in the period between March 2021 and February 2022 (1 year). The VOCs circulating in Calabria were first identified by Sanger sequencing of the S gene and then accurately genotyped by whole-genome NGS sequencing of representative isolates.

The main findings of this study were as follows: (i) The characterization of the dynamics of viral infection in the Calabria region, (ii) the description of the geographic distribution of mutations identified in the sequenced viral genomes of patients from Calabria, (iii) the identification of mutations predictive of reduced binding to class I MHC and/or reduced recognition by T cells, and (iv) the characterization of the effects of the most common mutations on S protein surface accessibility and the strength of the Spike–ACE2 interaction.

In regard to the dynamics of the viral infection in the Calabria area, our results indicate that B.1 and Alpha (26 and 74%, respectively) were the only circulating variants in Calabria in March 2021, when the study started, while a progressive decrease of B.1 cases was observed in the following months. Conversely, Alpha remained predominant until June 2021, which was partly consistent with the dynamic of infection observed in Italy (ISS, 2021a) (first reported in North Italy in February 2020; predominant until October 2020).^3^ All sequenced B.1 samples identified in this study presented the D614G mutation in the S protein. The 614G variant emerged in February 2020 and overtook the 614D virus within 3 months (Korber et al., 2020) and represent the first dominant mutation associated with increased transmissibility of SARS-CoV-2 observed during the first wave of pandemic (Kwarteng et al., 2021; Wang et al., 2021a).

The presence of additional mutations (i.e., S477N, N439K-Δ H69/ ΔV70, and Q675H-A222V) was used to define specific B.1 sub-genotypes. Mutation S477N was first identified in the United States of America as a subtype of Iota and has occurred multiple times in Europe (Hadfield et al., 2018) including in Calabria in March 2020 (this study), identifying a sub-group of B.1 patients (B.1_ S477N). Mutation S477N has been associated with increased affinity for ACE2 receptor, increased transmissibility (Zahradník et al., 2021), and partial resistance to monoclonal antibodies (Liu et al., 2021). It is of interest that S477N, along with mutations E484K and P681H, is present also in other variants such as Kappa and Omicron.

The second B.1 sub-genotype was characterized by the simultaneous presence of N439K and ΔH69/ΔV70 (B.1_N439K_ΔH69/ΔV70). Mutation N439K emerged in Scotland in March 2020, increases binding affinity for ACE2 receptor and facilitates escape from neutralizing antibodies (Thomson et al., 2021). According to our data, the first evidence of N439K in Calabria area dates back to March 2020.

Also, ΔH69/V70 is a 6-nucleotide deletion (nt 21765–21770) that was detected on a D614 background in January 2020 in the United States of America and Thailand, and on a G614 background in April 2020 in Sweden (Meng et al., 2021). Moreover, ΔH69/V70 deletion has been observed in Alpha and Omicron (Mccarthy et al., 2021). The apparent impact of ΔH69/V70 on the epidemiological characteristics of SARS-CoV-2 is to increase infectivity but not immune evasion (Meng et al., 2021). The prevalence of ΔH69/V70 in Italy has increased since August 2020. According to our data, the first identification of ΔH69/V70 in Calabria area is of March 2021.

The third B.1 sub-genotype was B.1_Q675H_A222V. Mutation Q675H is considered an immune escape mutation that may affect the efficacy of vaccines (Li et al., 2020) that first appeared in the B.1.28 lineage in Montevideo (Uruguay) in November 2020. Our study reports the first identification of Q675H in Calabria in March 2021. Mutation A222V is known to impair virus entry (Hodcroft et al., 2021). It appeared in Spain (variant 20A.EU1) in early Summer 2020, spreading subsequently all over Europe (Mccallum et al., 2021). Our study reports the first identification of A222V in Calabria in March 2021.

During the second wave of pandemic, a limited number of variants emerged worldwide, namely, Alpha, Beta, and Gamma. Since their appearance, these variants displayed high transmissibility and therefore were categorized as VOCs.

In Italy, Alpha was first reported in October 2020 and became dominant until mid-June 2021. The first report of Alpha variant in Calabria is of March 2021, in the province of Reggio Calabria (data from this study). We found that its regional prevalence was very high at the end of Winter 2020–2021 and during Spring 2021 (74, 90, 73, and 87 in the period March–June 2021, respectively), rapidly declining thereafter (46% in July 2021 and 1.3% in August 2021). Accordingly, ISS reports indicated a similar trend of diffusion of Alpha in Italy (87% in March 2021, 92% in April 2021, 88% in May 2021, and 0.9% in June 2021).

All Alpha samples sequenced in this study had a similar molecular pattern that included, among others, ΔH69/V70, N501Y, A570D, D614G, and P681H. Mutation N501Y has occurred independently in the United Kingdom (Alpha), South Africa (Beta), and Brazil (Gamma). Consistent with its convergent evolution, N501Y represents a critical determinant of enhanced infection and increased transmission (Liu et al., 2021). Notably, the frequent co-occurrence of ΔH69/V70 deletion with N501Y suggests a possible cooperative contribute to increased transmissibility (Tian et al., 2021).

The results shown in this study also indicated that Alpha patients presented additional mutations in the S protein such as A701S, L5F, and T724I. Mutation L5F, observed in three patients of this study in March 2021, was first observed in Iota in New York, along with other common mutations (West et al., 2021). It is apparently responsible for increased infectivity due to enhanced folding, assembly, and secretion. Mutation T724I, observed in four patients of this study in March 2021, has been shown to increase the stability of Spike, facilitating the interaction with ACE2 receptor (Teng et al., 2021). Finally, T95I, observed in one Alpha patient in March 2021, was first identified in the Iota variant sequenced in New York, early in 2021 and then identified in AY.4.2 sub-lineage of Delta and in Omicron (Mccallum et al., 2021; Zhang et al., 2022).

At the same time, Gamma, a variant identified in Brazil in December 2020, appeared in Calabria in May 2020, reaching a frequency of 21% and declining shortly thereafter. In agreement with these results, Gamma was identified in Italy in February 2021, reaching a peak of 12% in June 2021. Gamma demonstrated immune escape from the neutralization of convalescent and vaccinated sera or neutralizing antibodies (Wang et al., 2021b). The observed immune escape was ascribed to the presence of E484K and N501Y. Also, P681H observed in Alpha and Gamma patients recruited in this study has been shown to increase transmissibility (Coutard et al., 2020; Tang et al., 2021).

Interestingly, in July 2021 a rare variant, named C.36.3, was identified in two patients from Reggio Calabria and in one patient from Catanzaro. The variant, C.36.3, a sub-lineage of C.36, appeared in Italy in May 2021. It was characterized by lower sensitivity to antibodies but similar affinity for ACE2 receptor (Castelli et al., 2021). In Italy, this variant was rare according to ISS reports, having been identified in few patients all over the country. To date 54 cases of C.36.3 have been registered, distributed among Emilia Romagna (*n* = 9), Liguria (*n* = 3), Lombardia (*n* = 23), Sardegna (*n* = 1), Sicilia (*n* = 7), and Veneto (*n* = 9), and, notably, in Calabria (*n* = 3 patients identified in this study). Among the mutations present in the C.36.3 genome, all patients presented Q677H, which will be shared with the Eta variant while one patient presented R346S, first identified in the Alpha variant and then typically associated with L452R in the Delta variant. Despite its limited effect on ACE2 binding, R346S has been classified as an immune-escape mutation (Yi et al., 2021). Interestingly, a different mutation occurring in the same residue 346 (R346K) has been identified in the Omicron variant.

At approximatively the same time, Delta became dominant in Italy. It was first identified in May in Veneto and Lombardia, rapidly spreading all over the country. From July 2021, it replaced Alpha. In June 2021, the B.1.617.2 sub-genotype became the dominant strain globally. Our results indicated that Delta was first identified in a patient from Reggio Calabria in June 2021 and became the only circulating VOC in Calabria from August to December 2021. The patients infected by Delta accrued in this study presented a common core of mutations in the genes encoding Spike, ORF3a, ORF7a, M and N proteins, being distributed among different AY sub-lineages (i.e., AY.4, AY.7.2, AY.43, AY.42, AY.61, and AY.122; Outbreak (Outbreak.Info, 2021a,b,c,d,e,f,g). In addition, several Delta isolates presented additional, mutually exclusive mutations in S protein, which allowed the definition of sub-genotypes Delta_T95I, Delta_A222V, and Delta_G181V (Table 2). Mutations A222V and T95I were more common in subvariants AY.4, AY.4.2, and AY.61 (Dubey et al., 2021).

It is of note that the different subtypes of the Delta variant present frequent relevant mutations also in proteins different from Spike such as I82T in the M protein, which is apparently specific of Delta (Suratekar et al., 2022). This mutation appeared in Italy in April 2021 and has risen to over 90% in December 2021. At the beginning of the pandemic, the mutation rate of M was very low, but it has increased over 100-fold in the last 4 months. This can be explained by an underlying biological advantage recently attributed to the ability of the M protein to inhibit the production of type I and III interferons (Zheng et al., 2020).

Finally, the Omicron variant appeared in Italy at the beginning of December 2021, and rapidly spread all over the country displacing Delta (ISS, 2021b). In Calabria, the first patient infected by Omicron was reported in the end of December 2021. Initially, Omicron coexisted with Delta throughout January 2022 (28 and 72%, respectively) as also indicated by ISS reports of January 2022 (3/01, 17/01, and 31/01) (ISS, 2021a).

The Omicron variant presents a large number of mutations, some of which have been reported in other VOCs such as S477N (B.1 variant); ΔH69/V70 and P681H (Alpha variant); H655Y (Gamma variant); K417N (Beta variant and AY variants); T478K (Delta variant); A701V (Beta and Iota variants); and R346K (Mu variant) (Suratekar et al., 2022). A notable mutation identified in the genomes of Omicron sequenced in this study occurs in residue E484, a key player in immune escape (Laurini et al., 2021). Accordingly, E484K has been described in a large number of variants such as Alpha, Eta, Gamma, Zeta, Theta, Beta, and Iota while E484Q and L452R were typical of Delta/Kappa lineage. The mutation identified in the Omicron genome in this study was a different one, namely, E484A.

A special note is for Q493R in the Spike protein, which was fixed in the genome of Omicron in April 2021, after treatment with bamlanivimab and etesivimab (Focosi and Maggi, 2021; Laurini et al., 2021), D3G, Q19E, and A63T in the M protein, which confer biological advantage to viral genomes.

The second point was the geographical distribution of the mutations identified in Calabria in this study. Our analysis indicated that (i) variants B1_S477N, Alpha_L5F, Delta_T95, Delta_G181V, and Delta_A222V were distributed all over Europe, (ii) variants B1_N439K_D69 and Delta_T95I were present in the majority of Italian regions, (iii) variants B1_S477N_T29I_E484Q, Alpha_A701S, and Alpha_T724I were present mainly in Calabria.

Mutation S477N was identified in many European countries, whereas the combination of S477N with T29I was registered almost exclusively in Italy. In fact, 23 out of 26 total cases were from Italy. Notably, all the Italian sequences present in the GISAID database in April 2022 were from the Calabria Region. Moreover, a sub-genotype of B.1 characterized by the simultaneous presence of S477N, T29I, and E484Q was exclusively detected in Italy (two cases in Calabria and one in Lazio) (COVID-Miner, 2020).

Conversely, the Alpha_A701S sub-genotype was detected in 11 European countries, though the majority of cases (64%) were from Italy. Notably, 158 out of the 172 isolates presenting A701S were detected in Calabria, with the remaining cases in Campania and Puglia. A701 is localized near the furin cleavage site of the S protein. Alanine replacement with valine at position 701 (A701V) originally appeared in Beta in South Africa in September 2020. Conversely, the replacement of alanine with serine (A701S) has been found only in a Delta sub-lineage from Sri Lanka (AY.28) (Outbreak.Info, 2021c).

Finally, the Alpha_T724I sub-genotype was detected in six European countries, although most cases (73%) were from Italy. Notably, 39 out of 40 isolates that presents T724I, which has been shown to increase the stability of the S protein (Teng et al., 2021), were detected in the Calabria area.

The third point was the analysis of the predicted effects of mutations on the immune response, including binding to class I MHC and recognition of T cells. The analysis performed in this study indicated that mutations identified in some residues of the S protein may be primarily responsible for impaired immune response. In particular, the mutations that predicted weaker or non-binding to class I MHC were T29I in B.1, A701S in Alpha and T19R in Delta. Moreover, mutations A67S present in Alpha, E156G, ΔF157/R158, and T478K present in Delta, R346S present in C.36.3, N439K, S477N, and E484Q present in B.1, R190S and E484K present in Gamma were predicted to induce decreased recognition of T cells (Guruprasad, 2022). However, also mutations in ORF7a protein, and in particular P68S present in the Delta variant, was apparently less efficiently recognized by T cells in comparison with its wild-type counterpart.

The amino acid substitutions identified in this study have also been analyzed in terms of exposure on the surface of the S protein and binding affinity for ACE2. The analysis reported here indicated that the residues within the RBD domain had the highest exposure values (E484, S477, and R346). This is consistent with the observation that some of the mutations that hit these residues (E484Q/K, S477I, and R346S) impair antibody recognition (Faria et al., 2021; Huang et al., 2021; Yi et al., 2021; Guruprasad, 2022). In addition, our results indicated that N439K, S477N, E484Q, and N501Y presented increased affinity for ACE2, in agreement with previous studies that have investigated the infectious potential of mutations in the S gene (Starr et al., 2020; Weisblum et al., 2020; Tsai et al., 2021; Zahradník et al., 2021; Chakraborty et al., 2022; Wrobel et al., 2022). On the contrary, K417N decreased ACE2 binding affinity though, in parallel, reduced also the neutralization potential of RBD-specific antibodies (Harvey et al., 2021; Laffeber et al., 2021).

In conclusion, we report on the results of SARS-CoV-2 surveillance performed in an area of South Italy (Regione Calabria), in the period between March 2021 and February 2022. The data reported here indicate that B.1 (26%) and Alpha (74%) were the only variants circulating in Calabria in March 2021, while Alpha became predominant in the following months until it was displaced by Delta in July 2021. Delta remained the prevalent VOC until December 2021, when it was replaced by Omicron. Notably, we identified sub-genotypes, enriched mainly in Calabria (B1_S477N_T29I_E484Q, Alpha_A701S, and Alpha_T724I), and characterized by mutations that can significantly impair binding to class I MHC molecules and virus recognition by T cells.

## Data Availability Statement

The data presented in the study are deposited in the GeneBank repository, accession numbers SUB11466277 and SUB11548142.

## Ethics Statement

The study involving human participants was reviewed and approved by Ethical Committee of Regione Calabria in the Meeting No. 434 held on December 16, 2021.

## Author Contributions

CDM and GV: conceptualization and design of the study. CDM, CV, AM, and MP: investigation and formal analysis. CDM, CV, AM, MP, FB, and GS: methodology, data curation, and visualization. NM, AQui, GSB, AG, LG, AGL, BQ, SS, AR, and ET: resources. DT, AQua, CT, GM, CDF, FSC, and GV: supervision, funding acquisition, and project administration. All authors contributed to manuscript revision, read, and approved the submitted version.

## Funding

This research received specific financial support from University Magna Graecia, Department of Experimental and Clinical Medicine (COVID19-DMSC) and Interdepartmental Center of Services (CIS), Molecular Genomics and Pathology, Magna Græcia University of Catanzaro, Italy. This work was also supported by Regione Calabria (noCOVID19@UMG POR Calabria – FESR/FSE 2014-2020 D.D.R.C. n. 4584 del 4/5/2021-Azione 10.5.12).

## Conflict of Interest

The authors declare that the research was conducted in the absence of any commercial or financial relationships that could be construed as a potential conflict of interest.

## Publisher's Note

All claims expressed in this article are solely those of the authors and do not necessarily represent those of their affiliated organizations, or those of the publisher, the editors and the reviewers. Any product that may be evaluated in this article, or claim that may be made by its manufacturer, is not guaranteed or endorsed by the publisher.

## Footnotes

^1^ECDC (2021). Available online at: https://www.ecdc.europa.eu/en/covid-19/variants-concern (accessed December 20, 2021).

^2^ISS (2020). Available online at: https://www.epicentro.iss.it/en/coronavirus/SARS-CoV-2-integrated-surveillance.

^3^Outbreak_Info (2021). Available online at: https://outbreak.info/situation-reports?pango=B.1.1.7 (accessed December 21, 2021).

## Supplementary Material

Supplementary Figure 1 Primers used to amplify S gene. S gene regions were amplified with the following indicated primers: SC2M1-55, SC2M1-56, SC2M1-57, SC2M1-58, SC2M1-59, SC2M1-60, SC2M1-61, and SC2M1-62b. SP, signal peptide; RBD, receptor-binding domain; FP, fusion peptide; HR1, heptad repeat domain 1; HR2, heptad repeat domain 2; TA, Transmembrane association domain; IT, intracellular terminal. Adapted from “An In-depth Look into the Structure of the SARS-CoV2 Spike Glycoprotein” by BioRender.com (2022). Retrieved from https://app.biorender.com/biorender-templates.

Supplementary Figure 2 Non-synonymous substitutions identified in SARS-CoV-2 genomes. The graph shows the number of non-synonymous substitutions among the genes of SARS-CoV-2 identified by NGS. Blue bars show the absolute number of substitutions/gene. Gray bars show the substitutions that have occurred in at least two isolates. ORF, open reading frame; S, Spike; E, envelope; M, membrane; N, nucleocapsid.

Supplementary Figure 3 Mutations in Gamma and C.36.3 isolates. Substitutions identified in the Gamma and C.36.3 isolates by NGS. Black, substitutions common to all Gamma isolates **(A)** and C.36.3 isolates **(B)**. ORF, open reading frame; S, Spike; E, envelope; M, membrane; N, nucleocapsid.
