# Supplementary figures and images for: Dynamics of Viral Infection and Evolution of SARS-CoV-2 Variants in the Calabria Area of Southern Italy
Source: Front Microbiol. 2022 Jul 28;13:934993. doi: 10.3389/fmicb.2022.934993 (PMC9366435; doi:10.3389/fmicb.2022.934993)

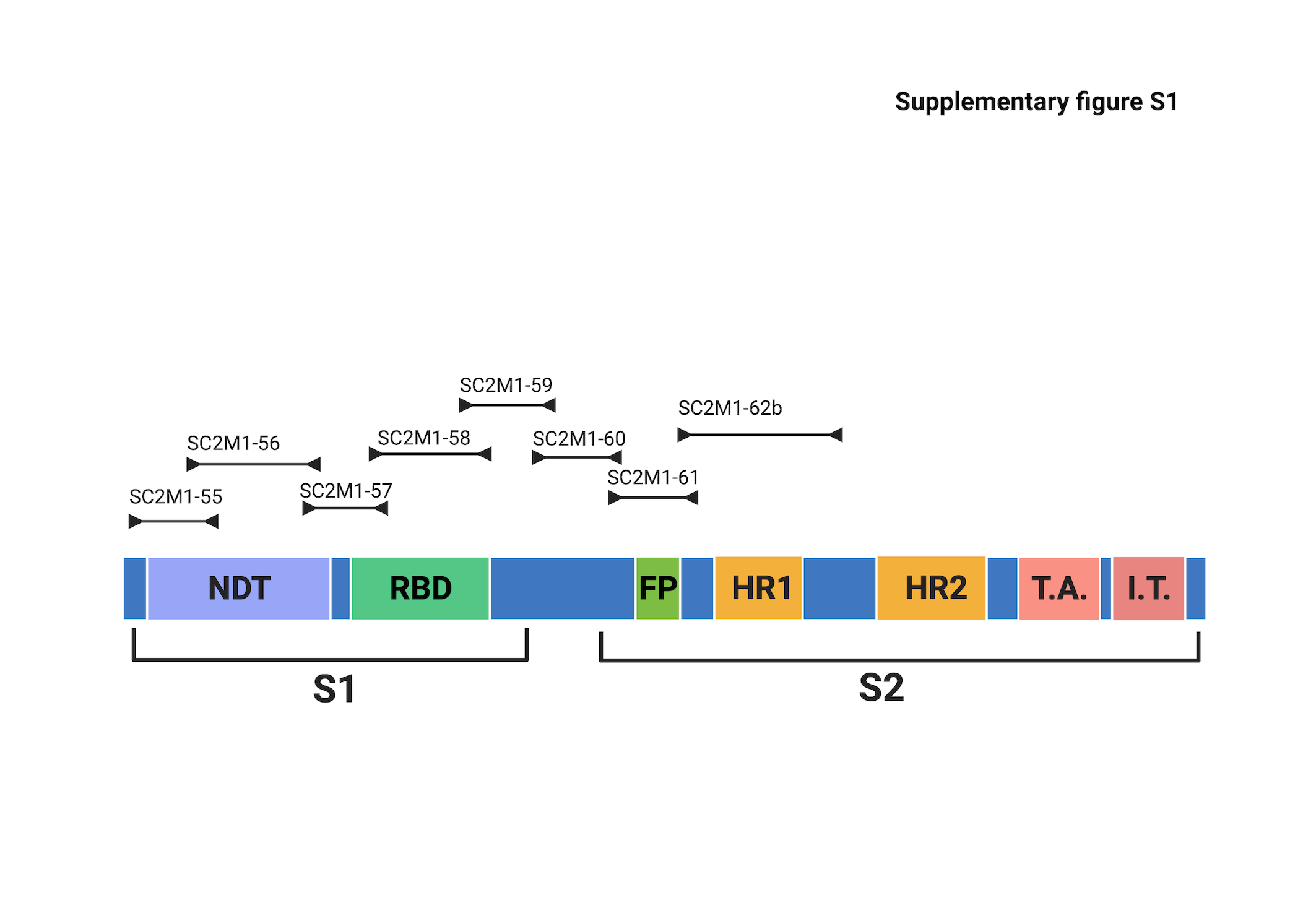

Supplement: Supplementary Figure 1 — Primers used to amplify S gene. S gene regions were amplified with the following indicated primers: SC2M1-55, SC2M1-56, SC2M1-57, SC2M1-58, SC2M1-59, SC2M1-60, SC2M1-61, and SC2M1-62b. SP, signal peptide; RBD, receptor-binding domain; FP, fusion peptide; HR1, heptad repeat domain 1; HR2, heptad repeat domain 2; TA, Transmembrane association domain; IT, intracellular terminal. Adapted from “An In-depth Look into the Structure of the SARS-CoV2 Spike Glycoprotein” by BioRender.com (2022). Retrieved from https://app.biorender.com/biorender-templates. [file Image_1.TIF]

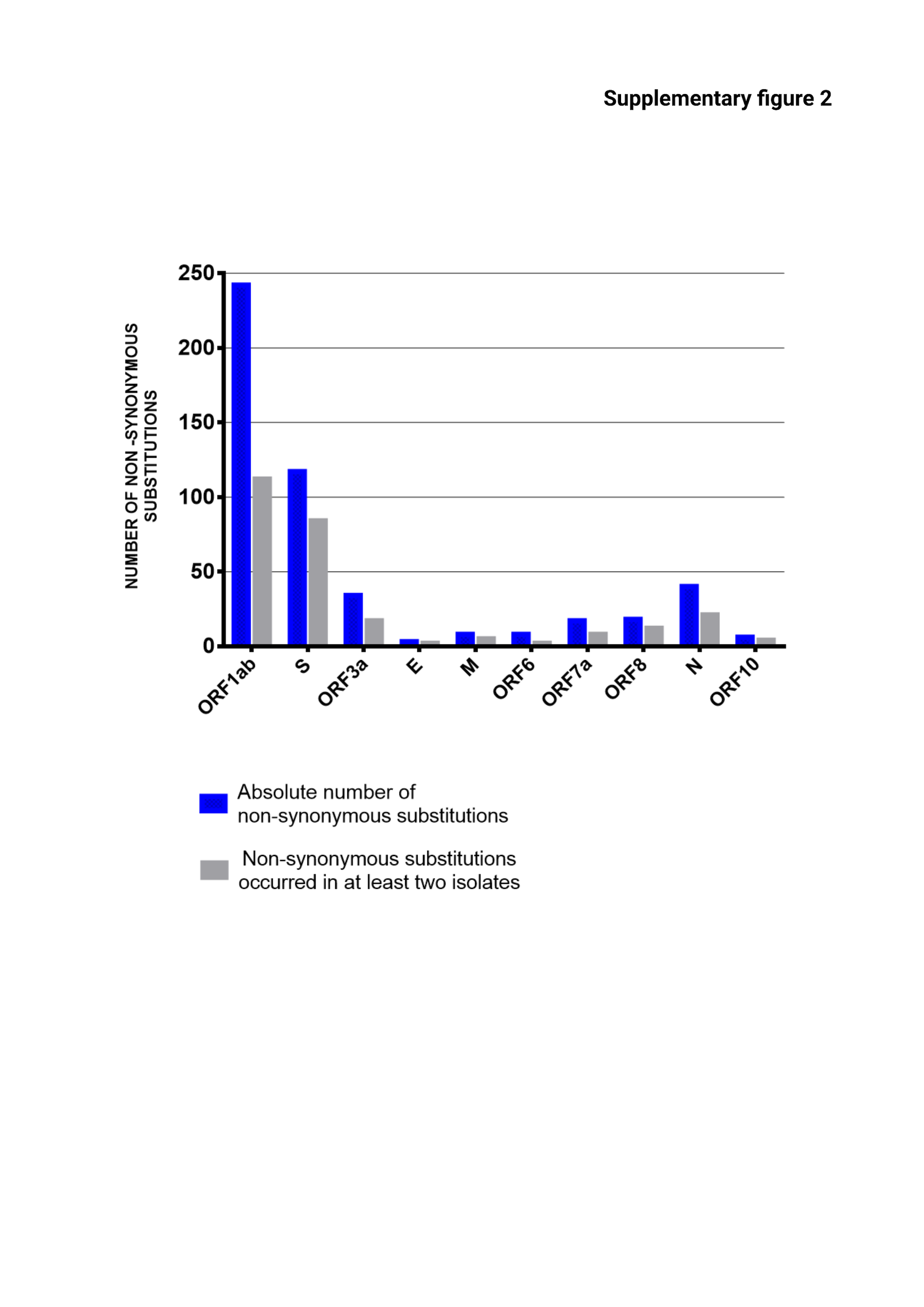

Supplement: Supplementary Figure 2 — Non-synonymous substitutions identified in SARS-CoV-2 genomes. The graph shows the number of non-synonymous substitutions among the genes of SARS-CoV-2 identified by NGS. Blue bars show the absolute number of substitutions/gene. Gray bars show the substitutions that have occurred in at least two isolates. ORF, open reading frame; S, Spike; E, envelope; M, membrane; N, nucleocapsid. [file Image_2.TIF]

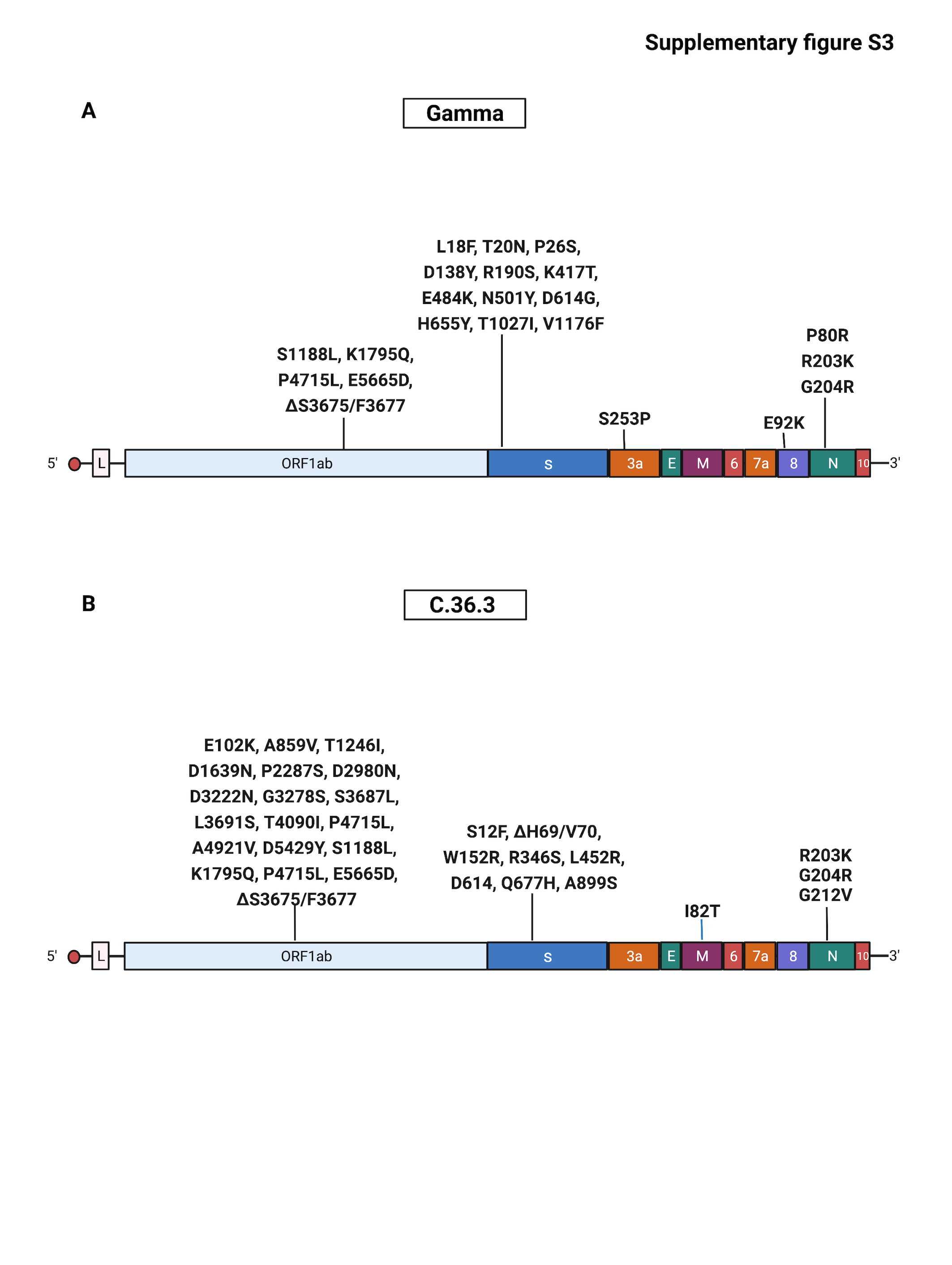

Supplement: Supplementary Figure 3 — Mutations in Gamma and C.36.3 isolates. Substitutions identified in the Gamma and C.36.3 isolates by NGS. Black, substitutions common to all Gamma isolates (A) and C.36.3 isolates (B). ORF, open reading frame; S, Spike; E, envelope; M, membrane; N, nucleocapsid. [file Image_3.TIF]
